# Paving the path for injury prevention in rugby‐7s: A systematic review and meta‐analysis

**DOI:** 10.1002/ejsc.12156

**Published:** 2024-06-27

**Authors:** Muhammed Rizaan Behardien, Janesh Ganda, Kathryn Dane, Stephen W. West, Carolyn A. Emery, Ben Jones, Sharief Hendricks

**Affiliations:** ^1^ Division of Physiological Sciences Department of Human Biology Faculty of Health Sciences University of Cape Town Cape Town South Africa; ^2^ Sports Rehab Centre Cape Town South Africa; ^3^ Wits Sport and Health (WiSH) School of Clinical Medicine Faculty of Health Sciences University of the Witwatersrand Johannesburg South Africa; ^4^ Discipline of Physiotherapy School of Medicine Trinity College Dublin Dublin Ireland; ^5^ Centre for Health, and Injury & Illness Prevention in Sport Department for Health, University of Bath Bath UK; ^6^ UK Collaborating Centre on Injury and Illness Prevention in Sport (UKCCIIS) University of Bath Bath UK; ^7^ Sport Injury Prevention Research Centre Faculty of Kinesiology University of Calgary Calgary Alberta Canada; ^8^ O'Brien Institute of Public Health University of Calgary Calgary Alberta Canada; ^9^ Hotchkiss Brain Institute University of Calgary Calgary Alberta Canada; ^10^ Alberta Children's Hospital Research Institute University of Calgary Calgary Alberta Canada; ^11^ McCaig Institute for Bone and Joint Health University of Calgary Calgary Alberta Canada; ^12^ Department of Community Health Sciences Cumming School of Medicine University of Calgary Calgary Alberta Canada; ^13^ Department of Pediatrics Cumming School of Medicine University of Calgary Calgary Alberta Canada; ^14^ Carnegie Applied Rugby Research (CARR) Centre Carnegie School of Sport Leeds Beckett University Leeds UK; ^15^ Premiership Rugby London UK; ^16^ England Performance Unit Rugby Football League Manchester UK

**Keywords:** fatigue, fitness, injury and prevention, team sport

## Abstract

This review and meta‐analysis aimed to describe the current rugby‐7s injury epidemiological literature by examining injury data from both sexes, all levels of play, and their associated risk factors. Studies published up until March 2024 were included. These studies were retrieved from six databases using search terms related to rugby‐7s or sevens, tackle, collision, collision sport, injury, athlete, incidence rate, mechanism, and risk factor. Only peer‐reviewed original studies using prospective or retrospective cohort designs with a clearly defined rugby‐7s sample were considered. Included studies needed to report one injury outcome variable. Non‐English and qualitative studies; reviews, conference papers, and abstracts were excluded. Twenty studies were included. The meta‐analysis used the DerSimonian–Laird continuous random‐effects method to calculate the pooled estimated means and 95% confidence interval. The estimated mean injury incidence rate for men was 108.5/1000 player‐hours (95% CI: 85.9–131.0) and 76.1/1000 player‐hours (95% CI: 48.7–103.5) for women. The estimated mean severity for men was 33.9 days (95% CI: 20.7–47.0) and 44.2 days (95% CI: 32.1–56.3) for women. Significantly more match injuries occurred in the second half of matches, were acute, located at the lower limb, diagnosed as joint/ligament, and resulted from being tackled. Fatigue, player fitness, and previous injuries were associated with an increased risk of injury. There were no statistically significant differences between women's and men's injury profiles. However, the inherent cultural and gendered factors which divide the two sports should not be ignored. The findings from this review will help pave the way forward beyond the foundational stages of injury prevention research in rugby‐7s.

## Highlights

- Rugby‐7s injury prevention research has not moved past the early stages of injury prevention research frameworks, such as the TRIPP model, because the injury epidemiological profile has not been comprehensively summarised.
- Significantly, more match injuries occurred in the second half, were acute, located at the lower limb, diagnosed as joint/ligament and resulted from being tackled. Fatigue, player fitness, and previous injuries were associated with an increased risk of injury. However, there were no differences across sex.
- These findings will help pave the way forward beyond the foundational stages of injury prevention research in rugby‐7s and will form the basis from which future injury prevention strategies can be developed.

## 1 INTRODUCTION

Rugby sevens (rugby‐7s) is a collision sport played by seven players over two seven‐minute halves, with laws similar to the fifteen‐a‐side version of the game. The international rugby‐7s competition is structured as an annual series. This usually consists of 10 men's tournaments and seven women's tournaments played over seven months. On five occasions, two consecutive tournaments are played within seven days of one another. Each tournament lasts for two to three days, with each team playing up to three matches per day (World Rugby, 2022). Rugby‐7s is played at high speeds with high‐intensity work rates, running manoeuvres and collisions (Cruz‐Ferreira et al., 2017; Dane et al., 2022; den Hollander, Lambert, et al., 2021; Ross et al., 2015). The congested schedule and match demands of rugby‐7s pose a unique set of challenges for injury prevention and management. Research has shown that across all sporting codes participating in the Olympic Games, rugby‐7s accounts for one of the highest injury incidence rates (18.6 injuries per 100 athletes) (Nabhan et al., 2016; Soligard et al., 2017; Steffen et al., 2020). Rugby‐7s has a higher injury incidence rate than rugby‐15s (101.5–119.8 injuries/1000 player‐hours vs. 89.1–96.0 injuries/1000 player‐hours) (Cruz‐Ferreira et al., 2017) as well as rugby league (87.7 injuries/1000 player‐hours) (King D et al., 2022) highlighting the increased injury risk associated with the sport. As such, World Rugby—the sport's governing body—recognises rugby‐7s injury prevention as a top research priority (Tucker, 2016).

Even though research on rugby‐7s has increased since its introduction into the Olympics in 2016, most of what is known about the sport's injury profile is based on injury surveillance studies (Burger et al., 2020). To our knowledge, there is also only one published systematic review focusing on rugby‐7s injury epidemiology (Cruz‐Ferreira et al., 2017). This review reports that at the men's international level, injury incidence rates ranged from 101.5 to 119.8 injuries/1000 player‐hours and severity from 34.1 to 51.5 days' time‐loss (Cruz‐Ferreira et al., 2017). The authors acknowledged that their review was limited by the inclusion of unpublished injury surveillance reports, cohorts reporting only men's injury data, and injury data from only one amateur cohort. Within injury prevention frameworks, such as the *‘Translating Research into Injury Prevention Practice’* (TRIPP) (see Supporting Information S1: 1) (Finch, 2006), the foundation always begins with a comprehensive understanding of the injury epidemiology profile and the associated injury risk factors. Due to the extant rugby‐7s literature, injury prevention research within this field has not progressed past the initial stages of the TRIPP model. This review thus aims to provide the foundational knowledge to move beyond the first stages of the TRIPP model in order to facilitate the development of evidence‐based, specific rugby‐7s injury prevention interventions. Therefore, this systematic review and meta‐analysis takes a more comprehensive and analytical approach to describe the current state of rugby‐7s injury epidemiology literature by being the first to investigate all levels of play, both sexes, and summarise potential risk factors associated with injuries in rugby‐7s.

## 2 METHODS

This review was conducted using the Preferred Reporting Items for Systematic Reviews and Meta‐Analyses statement (Page et al., 2021). The study protocol was also prospectively registered on the Open Science Framework (OSF) Registries (registration DOI: https://doi.org/10.17605/OSF.IO/Z65QR).

### 2.1 Ethnicity, diversity and inclusion statement

Our research and author team included five men and two women, from different ethnicities and included both senior and early career investigators with various specialities within sports medicine. The team also included authors from various marginalised groups (people of colour, members of the LGBTQIA2S+ community, and people from low‐/middle‐income countries). The diversity of the research and author team was not prospectively determined.

### 2.2 Search strategy

A comprehensive search of PubMed; SCOPUS; Web of Science and MEDLINE Complete, CINAHL Complete, and HealthSource (via EbscoHost) was conducted in March 2024, with no date restrictions using the following search terms for rugby‐7s (sevens OR 7s OR rugby‐7s OR Sevens World Series (SWS) OR tackle OR collision OR collision sport) AND (injur* OR athlete* OR athletic injur* OR mechan* OR risk OR factor) OR (epidemiol* OR incidence OR rate). All full searches can be seen in Supporting Information S1: 2. Search filters were enabled for peer‐reviewed articles published within journals and to exclude conference abstracts and book chapters where possible. The reference lists of included studies were also scanned for any relevant, published, and peer‐reviewed literature.

### 2.3 Eligibility criteria

Similar to previous systematic reviews in rugby union and rugby sevens (Cruz‐Ferreira et al., 2017; Williams et al., 2021), the inclusion criteria were: (1) original research studies; (2) using a prospective or retrospective design; (3) with a clearly defined rugby‐7s sample; and (4) studies including at least one of the following injury epidemiological variables (Cruz‐Ferreira et al., 2017; Williams et al., 2013, 2021):

a) injury incidence rates for match and/or training,
b) injury incidence rates for women and/or men,
c) differences in injury incidence rates or proportion between backs and forwards,
d) mean and/or median injury severity,
e) injury location,
f) injury type or system injured,
g) period of the match the injury occurred,
h) what the injury‐causing event was,
i) if the injury was acute or chronic in nature; or
j) new or recurrent.

The exclusion criteria included: (1) articles not in English; (2) rugby league sevens samples; (3) studies not differentiating between the various rugby codes; (4) qualitative studies; and (5) reviews, abstracts, and conference abstracts that did not have links to full‐text papers.

### 2.4 Selection process

Duplicates were initially automatically removed by EndNote 20 (The EndNote Team, Philadelphia, USA, 2013). However, manual duplicate removal also had to be done by MRB. Thereafter, MRB screened the titles for eligibility. Abstracts were then excluded based on the inclusion/exclusion criteria. Full‐text articles that met the criteria were then retrieved. Full‐text screening was completed by two independent reviewers (MRB and JG). Disagreement was resolved by discussion until a consensus was reached.

### 2.5 Assessment of methodological quality and risk of bias

Joanna Briggs Institute (JBI) Critical Appraisal Tools are recommended as a set of quality assessment tools most applicable to the widest range of study designs (Ma et al., 2020). The ‘JBI Critical Appraisal Checklist of Studies Reporting Prevalence Data’ has also been reported to be used as a tool to assess the risk of bias (Glasgow et al., 2020). This JBI critical appraisal checklist was applied to all prospective and retrospective epidemiological and cohort studies reporting incidence rates and/or proportions. Studies were rated on nine criteria (see Supporting Information S1: 3). Responses are captured as “yes”, “no”, “unclear”, or “not appropriate”. JBI recommends that this tool not be totalled into a percentage score of quality.

### 2.6 Data extraction

MRB extracted the data from 100% of the included studies and KD extracted the data from 10% of the studies. The data were cross‐checked, where MRB checked KD's data extractions for accuracy and vice versa. Descriptive variables included: country and competition; level of play; study duration; and number of athletes included. Injury epidemiological variables that were extracted included: injury definitions, training and match exposure, injury incidence rate, injury severity, injury burden, location, injury type, mechanism of injury as well as player position, phase of play, and risk factors associated with injuries. Even though investigating concussive injuries was not a primary aim of this study, when concussion injury data were available, it was also extracted. This data extraction was guided by the consensus statement on injury definitions and data collection procedures for studies of injuries in rugby union (Fuller et al., 2007). Fuller et al. (2007) defined the injury incidence rate as the number of injuries per 1000 player‐hours; injury severity as the number of days absent from practice or competition; and Bahr et al. (2020) defined injury burden as the injury incidence rate multiplied by the mean severity of the sample. Data regarding injury location, type, and potential risk factors (incidence rates related to the injury, match half, and player position) were also extracted. Both time‐loss and medical attention injuries were included in the data. Time‐loss injuries are defined as any injury that results in absence for 24 h or longer from training or competition, whereas medical attention injuries are defined as an injury that requires medical assistance but does not result in any playing time being lost (Bahr et al., 2020).

### 2.7 Synthesis methods

Level of play was categorised according to: age, team representation (local, regional, and national), and ranking. ‘Age’ classified teams as either adolescent (U19 teams and younger) or senior (all adult teams) teams. The ‘team representation’ category was split into: local—schools, clubs and universities; regional—provincial, state, and regional representative teams; and national—teams representing their country in the first, second/B, or development teams. The ‘ranking’ category was based on the country's International SWS Rankings (World Rugby, 2022) where the respective teams are based. ‘Level 1’ was categorised as any first team belonging to a country ranked in the top 10 on the world rankings. Whereas, ‘level 2’ was categorised as any first team belonging to a country in the bottom half of the world rankings or any second/B team belonging to countries in the top 10 of the world rankings. Level 3 was classified as any classification not mentioned above. Teams were classified according to the world ranking of the year(s) the study was conducted. This was done because the SWS rankings change from season to season.

An attempt was made to calculate missing or inconsistently reported data based on other variables reported within the respective studies. For example, the absolute number of injuries per injury type was calculated when the total number of injuries and proportions per injury type were reported. When data were missing, a ‘not reported’ (*N/R*) was indicated in the results table. When the data category did not apply to a study, not applicable (N/A) was indicated. (e.g., the number of medical attention injuries in a study only reporting time‐loss injuries).

### 2.8 Statistical analysis

Where possible, all data are reported as means and/or percentages with their corresponding 95% confidence intervals (CIs). OpenMeta[Analyst] (Brown University, Rhode Island, USA, 2012) was used to conduct meta‐analyses where sufficient data were available. Meta‐analyses were only conducted if two or more datasets were available. The forest plots produced for these meta‐analyses were presented using the pooled estimated means and 95% CI. Note that the calculations embedded in OpenMeta[Analyst] software altered some of the data, and thus, the exact means and CIs may differ slightly from those reported in Table 1 and Supporting Information S1: 4. The DerSimonian–Laird continuous random‐effects analysis method was used to calculate the pooled estimated means and 95% CI for the available datasets (Paul et al., 2022). The *I* ^2^ statistic was also calculated to understand the heterogeneity of the data. *I* ^2^‐heterogeneity of 25%, 50%, and 75% were classified into arbitrary categories of low, moderate, and high, respectively (Higgins et al., 2003). The *I* ^2^ statistic indicates the amount of variance in the data due to the sampling error as compared to true chance. Across all the meta‐analyses, there were high levels of heterogeneity. Therefore—as mentioned above—the random‐effects model was used. This helped account for the differences between the studies as well as the use of several data entries from the same study (Williams et al., 2021). When meta‐analysed data were compared, the conservative CI‐overlap method was used as outlined by Sainani (2011) and Schenker and Gentleman (Schenker et al., 2001). Only when the CI of two datasets did not intersect, were they classified as statistically significant.

**TABLE 1:** Match data extracted from selected studies.

Reference	Setting	Level of play	Injury definition	Surveillance period	Sample	Sample injured (proportion, %)	Injury count	Total exposure (hrs)	Overall incidence rate (inj/1000 ph, 95% CI)	Time loss injuries (proportion, %)	Medical attention injuries (proportion, %)	Mean severity (days, 95% CI)	Injury burden
Senior national teams													
Fuller et al., 2010	HSBC SWS–All teams	National, level 1	Fuller, 2007	2008–2009	Men = 264	N/R	104	979.1	106.2 (87.8–128.9)	100%	N/A	45.0	4779.0
Fuller et al., 2015a ^b^	HSBC SWS–All teams	National, level 1	Fuller, 2007	2008–2013	Men = 1128	34 (3%)	34	4086.0	8.3 (5.9–11.6)	100%	N/A	19.3 (14.8–23.6)	160.2
Fuller et al., 2015b	HSBC SWS—All travelling teams	National, level 1	Fuller, 2007	2009–2014	Men = 563	N/R	319	2511.9	127.0 (113.8–141.7)	100%	N/A	48.5 (42.0–55)	6159.5
	HSBC SWS–All teams	National, level 1	Fuller, 2007	2009–2014	Men = 563	N/R	436	3363.2	129.6 (118–142.4)	100%	N/A	N/R	N/R
Soligaard et al., 2017	2016 Rio Olympics–All teams	National, level 1 + 2	Junge et al., 2008	Rio 2016	Women = 144	22 (15.3%)	22	N/R	15.3*/100 athletes*	5	N/R	N/R	N/R
					Men = 147	31 (21.1%)	31	N/R	21.1*/100 athletes*	4	N/R	N/R	N/R
					Overall = 291	54 (18.6%)	54	N/R	18.6*/100 athletes*	25 (46.3%)	29 (53.7%)	N/R	N/R
Fuller et al., 2016	HSBC SWS—All teams	National, level 1	Fuller, 2007	2008–2015	Men = 1816	702	N/R	6480.5	108.3 (100.6–116.6)	100%	N/A	44.2 (40.6–48.1)	4786.9
Fuller et al., 2017	HSBC SWS–All teams	National, level 1	Fuller, 2007	2014–2015	Women = 197	N/R	58	655.2	88.5 (68.4–114.5)	100%	N/A	42 (26–50)	3717.0
					Men = 331	N/R	135	1253.9	107.7 (90.9–127.4)	100%	N/A	28 (22–33)	3015.6
	HSBC SWS–All teams	National, level 1	Fuller, 2007	2015–2016	Women = 221	N/R	56	511.7	109.4 (84.2–142.2)	100%	N/A	30 (22–38)	3282.0
					Men = 340	N/R	153	1394.2	109.7 (93.7–128.6)	100%	N/A	21 (17–26)	2303.7
	Rio Olympics 2016–All teams	National, level 1 + 2	Fuller, 2007	Rio 2016	Women = 148	N/R	8	112.5	71.1 (35.6–142.2)	100%	N/A	92.0 (22.5–161.5)	6541.2
					Men = 152	N/R	14	112.5	124.5 (73.7–210.2)	100%	N/A	86 (38.4–133.6)	10707.0
Fuller, 2018	HSBC SWS–All teams	National, level 1	Fuller, 2007	2008–2016	Men	N/R	855	7875.0	108.6 (101.6–116.1)	100%	N/A	44.2 (40.9–47.8)	4800.1
Senior national teams													
Toohey et al., 2019 ^a^	HSBC SWS–Australian national teams	National, level 1	Fuller, 2007	2015–2017	Women = 35	27 (77.1%)	152	N/R	40.8 (34.5–47.8)	120 (78.9%)	32 (21.1%)	13 (IQR 46) *Median*	N/R
					Men = 55	46 (83.6%)	213	N/R	45 (39.2–51.5)	169 (74.6%)	44 (20.7%)	14 (IQR 29) Median	N/R
					Overall = 90	73 (81.1%)	365	8475	43.2 (38.8–47.8)	289 (79.2%)	76 (20.8%)	13 (IQR 34) Median	N/R
Fuller & Taylor, 2020	HSBC SWS–All teams	National, level 1	Fuller, 2007	2008–2019	Men = 3242	N/R	1461	11934.0	122.4 (116.3–128.9)	100%	N/A	43.0 (40.3–45.7)	5263.2
Fuller & Taylor, 2021	HSBC SWS–all teams	National, level 1	Fuller, 2007	2013–2020	Women = 1562	N/R	416	3938.0	105.6 (96.0–116.3)	100%	N/A	53.4 (46.9–59.9)	5639.0
Xu et al., 2021	Singapore Cricket Club National Rugby Sevens Tournament	National, level 2 + 3	Fuller, 2007 + Bahr et al., 2020	2012–2017	Men = 1380	N/R	343	985.6	348.0	N/R	N/R	N/R	N/R
Senior regional teams													
Cruz‐Ferreira et al., 2018	Portuguese National Rugby Sevens Circuit	Regional, level 2 + 3	Fuller, 2007 + 2010	2015–2016	Men = 226	N/R	27	201.6	133.9 (90.1–192.2)	100%	N/A	22.2 (10.1–40.8)	2972.6
		Regional, level 2	Fuller, 2007 + 2010	2015–2016	Men = 142	N/R	19	161.0	118.0 (73.1–180.9)	100%	N/A	28.0 (10.7‐55.4)	3298.1
		Regional, level 3	Fuller, 2007 + 2010	2015–2016	Men = 84	N/R	8	40.6	197.0 (91.5–374.2)	100%	N/A	8.6 (6.1‐11.0)	1700.1
Reis et al., 2018	North Circuit Rugby 7s–Mauna Stage, Brazil	Regional, level 2	Not specified	2016	Women = 39	14 (35.9%)	N/R	N/R	N/R	N/R	N/R	N/R	N/R
					Men = 26	4 (15.4%)	N/R	N/R	N/R	N/R	N/R	N/R	N/R
					Overall = 65	18 (27.7%)	N/R	N/R	N/R	N/R	N/R	N/R	N/R
Senior local teams													
Lopez et al., 2012	Local Area Union Rugby Sevens–USA	Local, level 1	Fuller, 2007	2010	Women = 658	N/R	N/R	N/R	10.0	N/R	N/R	N/R	N/R
					Men = 878	N/R	N/R	N/R	74.7	N/R	N/R	N/R	N/R
					Overall = 1536	48	48	866.3	55.4 (42.3–68.5)	75%	25%	N/R	N/R
Rizi et al., 2017	University Rugby–Hong Kong	Local, level 2	Fuller, 2008	2014–2015	Overall = 104	NR	16	270.0	59.3	100%	N/A	25.5 (SD +/− 23.2)	1510.8
Adolescent national teams													
Nabhan et al., 2016 ^a^	2014 Youth Olympic Games Nanjing–team USA	Adolescent national, level 1 + 2	Junge et al., 2008	2014 Youth Olympics	Women = 12	8 (66.7%)	N/R	N/R	N/R	100%	N/A	N/R	N/R
					Men = 12	4 (33.3%)	N/R	N/R	N/R	100%	N/A	N/R	N/R
					Overall = 24	12 (50%)	N/R	N/R	291.7*/1000 athletes*	100%	N/A	N/R	N/R
Steffen et al., 2020 ^a^	Youth summer Olympics Buenos Aires	Adolescent national, level 1 + 2	Junge et al., 2008	Buenos Aires 2018	Women = 71	26 (36.6%)	26	N/R	36.6*/100 athletes*	N/R	N/R	N/R	N/R
					Men = 72	35 (48.6%)	35	N/R	48.6*/100 athletes*	N/R	N/R	N/R	N/R
					Overall = 143	61 (42.7%)	61	N/R	42.7*/100 athletes*	22 (36.1%)	39 (63.9%)	N/R	N/R
Adolescent regional teams													
Lopez et al., 2020	USA U19 Rugby Sevens Tournaments^c^	Adolescent Regional, level 1 + 2	Fuller, 2007	2010–2014	61 women's teams	39	39	454.0	85.9	N/R	N/R	N/R	N/R
					256 men's teams	134	134	1646.0	81.4	N/R	N/R	N/R	N/R
					Overall = 3804 players	173 (4.5%)	173	2101.0	82.4	69 (39.9%)	104 (40.1%)	35.5	2925.2
Mixed teams													
Lopez et al., 2016 ^b^	Multiple USA Rugby‐7s Tournaments^d^	Regional level 1 + 2 and local level 1 + 2	Fuller, 2007 + Aubrey et al., 2001	2010–2013	Women = 3876	21 (0.5%)	21	N/R	8.1	100%	N/A	36.7 (20.8–52.6)	297.3
					Men = 9786	46 (0.5%)	46	N/R	7.6	100%	N/A	27.9 (20.3–35.7)	212.0
					Overall = 13644	67 (0.5%)	67	8672.0	7.7	100%	N/A	30.6	235.6
Ma et al., 2016 ^a^	Multiple USA Tournaments^e^	Regional level 1 + 2 and local level 1 + 2	Fuller, 2007 + Fuller, 2008	2010–2013	Women = 3876	N/R	253	2590.8	46.3 (38.4–55.4)	47.3%	43.7%	45.9 (33.1–58.7)	2125.2
		Regional level 2 and local, level 1 + 2	Fuller, 2007 + Fuller, 2008	2010–2013	Women = 3324	N/R	N/R	2130.8	49.3	105 injuries	N/R	41.8	2060.7
		Regional level 1	Fuller, 2007 + Fuller, 2008	2010–2013	Women = 552	N/R	N/R	460.0	32.6	15 injuries	N/R	74.9	2441.7

Abbreviations: N/A, not applicable to the study; N/R, not reported within the study; ph, player‐hours.

^a^ Both match and training data.

^b^ Only reported on concussion injuries.

^c^ Tournaments include: USA Rugby (USAR) Geographic Union (GU) 7s Series, USAR Territorial Union (TU)/Competitive Region (CR) Qualifier Rugby 7s Championship Series, and USA 7s Invitational.

^d^ Tournaments include: USAR Local Area/GU 7s Series, TU Qualifier Rugby 7s Championship Series, National Club 7‐a‐side Championship Series, All‐Star Championships, USA 7s LLC, USA 7s Invitational, and Collegiate Rugby Championships.

^e^ Tournaments include: USAR GU 7s Series, TU/CR Qualifier Rugby 7s Championship Series, National Club 7‐a‐side Championship Series, All‐Star Championships, USA 7s Invitational, and USA 7s High School Rugby Challenge.

## 3 RESULTS

**FIGURE 1:**
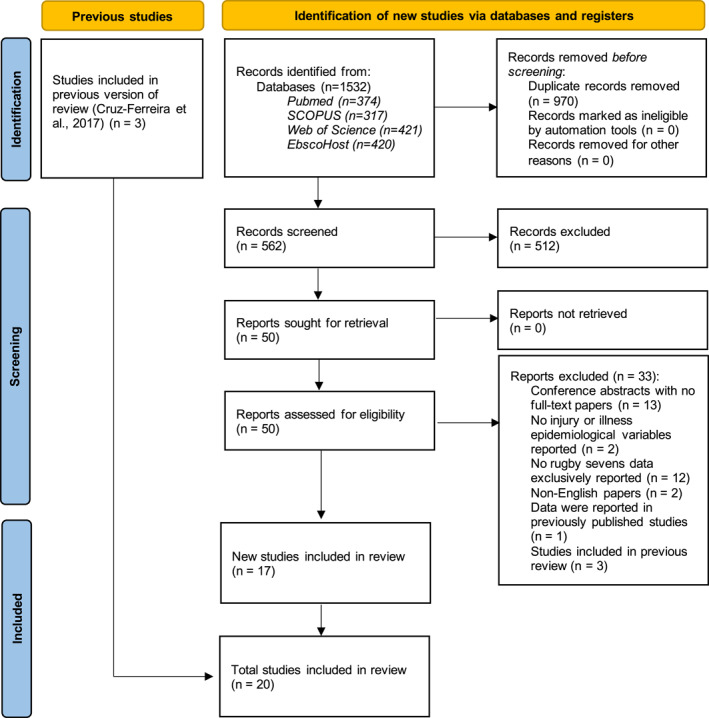
PRISMA literature retrieval and selection.

The initial search retrieved a total of 1532 studies. After duplicates were removed, the titles of the remaining 562 studies were screened, followed by the eligibility criteria being scrutinised within the abstracts. Thereafter, a further 512 studies were excluded. After 50 full‐text studies were reviewed by two researchers (MRB and JG), 33 studies were consequently excluded, and three additional studies were included as a result of manual citation searching. Twenty studies were included in the final review for full data extraction, three of which were included in the previously published systematic review (see Figure 1).

### 3.1 Description of studies

Seventeen studies used prospective and three studies used retrospective study designs. Eleven studies included national teams, and two studies included regional, local and national adolescent teams. One study also reported on regional adolescent teams, while another two studies consisted of mixed cohorts. Five studies included the USA teams (Lopez et al., 2012, 2016, 2020; Ma et al., 2016; Nabhan et al., 2016), and other studies included team(s) from Australia (Toohey et al., 2019), Brazil (Reis et al., 2018), Portugal (Cruz‐Ferreira et al., 2018), and Hong Kong (Rizi et al., 2017). Eleven studies reported both women's and men's injury data (Fuller et al., 2016, 2017; Lopez et al., 2012, 2016, 2020; Nabhan et al., 2016; Reis et al., 2018; Rizi et al., 2017; Soligard et al., 2017; Steffen et al., 2020; Toohey et al., 2019), whereas seven reported only men’s (Cruz‐Ferreira et al., 2018; Fuller, 2018; Fuller et al., 2010, 2015a, 2015b, 2020; Xu et al., 2021) and two reported only women's injury data (Fuller et al., 2021; Ma et al., 2016). The sample sizes of the selected articles ranged from 24 to 13,644 players (see Table 1), which resulted in a total of 10,839 women's and 20,418 men's players being included in this review. Two studies did not specify the number of women's and men's players (both = 3908 players). Therefore, a total of 35147 players were exposed to a total of 66858 match playing hours and sustained a total of 5137 match injuries between 2008 and 2020 across all included studies.

### 3.2 Assessment of methodological quality

The results from the quality assessment can be viewed in Supporting Information S1: 3. Sixteen studies (Cruz‐Ferreira et al., 2018; Fuller, 2018; Fuller et al., 2010, 2015a, 2015b, 2016, 2017, 2020, 2021; Lopez et al., 2012, 2016, 2020; Ma et al., 2016; Soligard et al., 2017; Steffen et al., 2020; Toohey et al., 2019) met the checklist criteria on seven or more occasions. Two received five (Nabhan et al., 2016; Rizi et al., 2017; Xu et al., 2021) and two (Reis et al., 2018) received four positive responses to the questionnaire. Those that answered met the checklist criteria on five or fewer occasions excluded reporting of how missing data or follow‐ups were handled; conducting and reporting appropriate statistical analyses; performing adequate stratified analyses of their cohorts; describing the subjects and setting in enough detail; and studying an adequate sample size.

### 3.3 Overall injury incidence rates

#### 3.3.1 Match injuries

**FIGURE 2:**
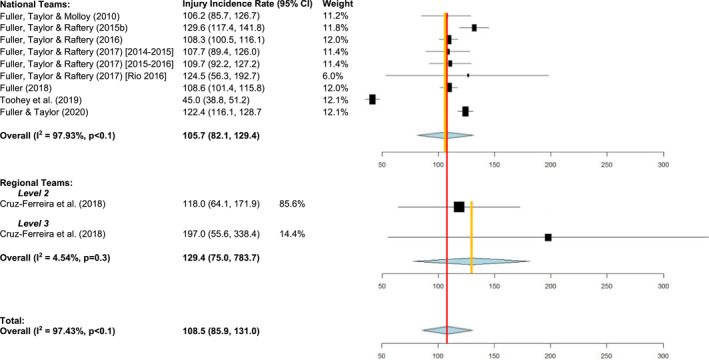
Injury incidence rates by the level of play for senior teams.

Seventy‐five percent (15/20) of the studies reported injury incidence rate as *injuries per 1000 playing hours*. Overall, the pooled estimated mean injury incidence rate for men was 108.5/1000 player‐hours (95% CI: 85.9–131.0; *I* ^2^ = 97.4% and *p* < 0.1) and 76.1/1000 player‐hours (48.7–103.5; *I* ^2^ = 96.3% and *p* < 0.01) for women. The pooled estimated match injury incidence rate for men's senior national teams was 105.7/1000 player‐hours (82.1–129.4; *I* ^2^ = 97.9% and *p* < 0.1) (see Figure 2). In men's senior regional teams, the injury incidence rate was equal to 133.9/1000 player‐hours (90.1–192.2) and 74.7/1000 player‐hours in men's senior local teams from the USA. Australian national men's teams had a significantly lower injury incidence rate (45.0/1000 player‐hours (38.8 and 51.2)) than all other studies as well as the pooled estimated match injury incidence rate. The pooled estimated match injury incidence rate for women's senior national teams was 83.1/1000 player‐hours (45.1–121.2; *I* ^2^ = 96.8% and *p* < 0.01) (see Figure 2). The only study investigating lower levels of play for women's teams reported an injury incidence rate of 46.3/1000 player‐hours (38.4–55.4) for regional and local teams in the USA (Ma et al., 2016). There were no significant differences found by the level of play within men's and women's teams' injury data, and no significant differences were found between sexes.

#### 3.3.2 Training injuries

Of the four studies that reported training injury outcomes, injury incidence rates were reported by 75% (3/4) of these studies. Training injury incidence rates ranged from 1.0/1000 player‐hours (0.4–2.1) to 2.2/1000 player‐hours (1.2–4.1) in women's senior national teams (Fuller et al., 2021) and 0/1000 player‐hours to 1.2/1000 player‐hours (0.7–2) in men's senior national teams (Fuller et al., 2017) (see Supporting Information S1: 4). Senior local teams reported an injury incidence rate of 3.3/1000 player‐hours (Rizi et al., 2017). No significant differences were found between sexes and level of play. However, the training injury incidence rate was significantly lower than the match injury incidence rate.

#### 3.3.3 Concussion

Concussion incidence rates ranged from 7.7/1000 player‐hours in a sample of regional and local senior USA teams (Lopez et al., 2020) to 8.3/1000 player‐hours in men's senior national teams (Fuller et al., 2015a). Within the USA sample, it was found that women's and men's concussion incidence rates were similar (women = 8.1/1000 player‐hours and men = 7.6/1000 player‐hours).

### 3.4 Injury severity

#### 3.4.1 Match injury severity

**FIGURE 3:**
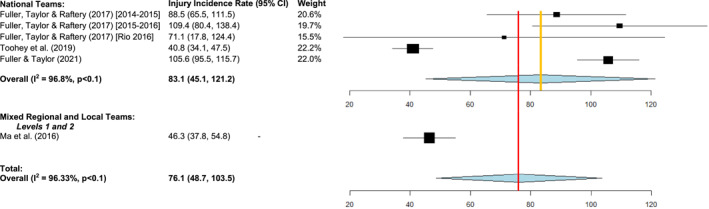
Injury severity by the level of play for senior teams.

Sixty‐five percent of studies (13/20) reported injury severity as the mean number of days absent from play. Six of these 13 studies also classified injury severity as outlined by Fuller et al. (2007): slight (0–1 day), minimal (2–3 days), mild (4–7 days), moderate (8–28 days), and severe (>28 days) (Fuller et al., 2010; Lopez et al., 2012; Ma et al., 2016; Rizi et al., 2017; Soligard et al., 2017; Steffen et al., 2020). Lopez et al. (2020) only reported injury severity using the Fuller et al. method (Fuller et al., 2007). One study reported severity as the median number of days absent from play (Toohey et al., 2019). Overall, the pooled estimated mean injury severity for men was 33.9 days (20.7–47.0; *I* ^2^ = 98.7% and *p* < 0.1) and 44.2 days (32.1–56.3; *I* ^2^ = 81.6% and *p* < 0.1) for women. The pooled estimated injury severity for men's senior national teams was 37.6 days (29.1–46.1; *I* ^2^ = 95.2% and *p* < 0.1) (see Figure 3). In men's senior regional teams, injuries resulted in a mean of 22.2 (10.1–40.8) days absent from play, and in senior local teams from Hong Kong, it resulted in 25.5 (SD ± 23.2) days. Within the regional teams, level 2 and level 3 teams sustained injury severities of 28.0 days (5.6–50.3) and 8.6 days (6.2–11.1), respectively. The only significant difference in men's injury severity was between national teams and regional level 3 teams (36.3 (25.8–46.9) versus 8.6 (6.2–11.1)). The pooled estimated injury severity for women's senior national teams was 44.1 days (28.6–59.5; *I* ^2^ = 86.2% and *p* < 0.01) (see Figure 3). The injury severity of women's regional and local teams from the USA was 45.9 days (33.1–58.7). No significant differences were noted across the level of play for women's teams. No significant differences were also found between sexes.

#### 3.4.2 Training injury severity

Of the four studies that reported training injury outcomes, injury severity was reported by 75% (3/4) of these studies. Women's senior national teams' injury severity ranged from 27.0 to 67.1 days and in men's senior national teams from 0 to 43.5 days. One men's senior local team study reported an injury severity of 8.6 days (SD: ±8). No significant differences were found between sexes or levels of play due to the lack of reporting of data dispersion.

#### 3.4.3 Concussion injury severity

Concussion injuries in men's senior national teams resulted in a mean of 19.3 days (14.8–23.6) missed from play (Fuller et al., 2015a). Whereas, the USA sample of regional and local teams was found to have a mean injury severity of 36.7 days (20.8–52.6) and 27.9 days (20.3–35.7) for women and men, respectively (Lopez et al., 2016) (see Supporting Information S1: 5). No significant differences were noted across levels of play or between sexes.

### 3.5 Injury burden

#### 3.5.1 Match injury burden

Sixty percent of the studies (12/20) reported both injury incidence rates and injury severity (as mean number of days), which made it possible to calculate injury burden (days/1000 player‐hours). The pooled estimated men's senior national teams' injury burden was 4986.9 days/1000 player‐hours. In men's regional teams, the pooled estimate was equal to 2972.6 days/1000 player‐hours. However, within the regional team classification, injury burden was reported for level 2 and 3 teams as 1700.1 days/1000 player‐hours and 3298.1 days/1000 player‐hours, respectively. A senior local cohort from Hong Kong which included both men's and women's teams resulted in an injury burden of 1510.8 days/1000 player‐hours (Rizi et al., 2017). The women's senior national teams' pooled estimate was 3702.5 days/1000 player‐hours. In a cohort of women's regional and local teams, the injury burden was equal to 2152.2 days/1000 player‐hours.

#### 3.5.2 Training injury burden

During the training, women's senior national teams had an injury burden of 101.9 days/1000 player‐hours and men's senior national teams had an injury burden of 38.8 days/1000 player‐hours. The only study to report on senior local teams had a training injury burden of 28.5 days/1000 player‐hours (Rizi et al., 2017).

#### 3.5.3 Concussion burden

Studies focusing on concussion injuries reported injury burdens ranging from 160.2 days/1000 player‐hours in men's senior national teams (Fuller et al., 2015a) to 235.6 days/1000 player‐hours in regional and local teams in the USA (Lopez et al., 2016). Within the USA cohort, women's and men's teams incurred an average injury burden of 297.0 days/1000 player‐hours and 212.0 days/1000 player‐hours, respectively.

#### 3.5.4 Injury burden as a function of injury type

Twenty percent of studies (4/20) reported the injury incidence rates and severity for different types of injuries (see Supporting Information S1: 6). These four studies reported on six different cohorts including national (Fuller et al., 2010, 2015b), regional, and local (Lopez et al., 2016) as well as adolescent teams (Lopez et al., 2020). Overall, bone injuries accounted for the greatest injury severity followed by muscle/tendon injuries. Joint/ligament injuries account for the highest injury incidence rate.

### 3.6 Injury location

Seventy‐five percent of the studies (15/20) that reported injury location data (see Supporting Information S1: 7) reported four main injury location categories: head/neck, upper limb, trunk, and lower limb. The location that accounted for the highest proportion of injuries was the lower limbs, except for men's senior local teams, which found that the upper limbs were most frequently injured (32.4%). Trunk injuries were found to have the lowest proportion of injuries across all cohorts. The significant differences noted across sex and the level of play were that the proportion of upper limb injuries in men's senior local teams (32.4% (28.7–36.1)) was greater than both women's and men's senior national teams' proportions (20.6% (15.7–25.4) and 19.9% (11.7–28.2)).

### 3.7 Injury type

#### 3.7.1 Match injuries

In all samples except adolescent national teams, the injuries with the highest proportion were joint/ligament and muscle/tendon injuries (see Supporting Information S1: 8). The injuries reported with the lowest frequencies were cartilage/impingement, joint dislocation, tendon, and other injuries. Men's senior regional teams' proportion of joint/ligament injuries (46.6% (41.7–51.6)) was significantly higher than men's senior national teams (27.8% (16.0–39.7)) and women's adolescent regional teams (25.5% (15.1–39.6)). Across all cohorts, significantly more injuries were classified as acute compared to gradual onset (92.6% (91.2–94.1) versus 7.4% (5.6–9.1)) (see Supporting Information S1: 9).

#### 3.7.2 Concussion

Eighty‐five percent of the studies (17/20) reported the proportion of concussions that were sustained (Fuller et al., 2010, 2015a, 2015b, 2016, 2017, 2020, 2021; Lopez et al., 2012, 2016, 2020; Ma et al., 2016; Nabhan et al., 2016; Rizi et al., 2017; Soligard et al., 2017; Steffen et al., 2020; Toohey et al., 2019; Xu et al., 2021). This included senior national and local teams as well as adolescent national and regional teams. No significant differences were noted across sex and level of play (see Supporting Information S1: 8).

### 3.8 Match injury event

Seventy‐five percent of the studies (15/20) reported the event associated with the injury (see Supporting Information S1: 10–12). All cohorts found that contact events (80.4% (76.1–84.6)) were associated with injury significantly more frequently than non‐contact events 18.6% (14.9–22.3) (see Supporting Information S1: 11). Overall, being tackled (33.9% (30.0–37.8)) followed by tackling (25.6% (19.4–31.8)) were the most frequently occurring events associated with injury (see Supporting Information S1: 10). The pooled estimated proportion for injuries occurring during the tackle event (tackling and being tackled) was significantly greater than all other injurious match events (58.3% (52.7–63.9)). Being tackled also accounted for a significantly greater proportion of injuries, compared to all other match events, except tackling. Lopez et al. (2020) and Cruz‐Ferreira (Cruz‐Ferreira et al., 2018) showed that incidental collisions (33.3% (13.1–53.5) and lineouts (33.3% (14.3–55.0)) were the second most injurious match activities in their respective studies. Across all cohorts, backs sustained a significantly higher proportion of the total injuries than forwards (62.4% (50.3–74.4) versus 36.8% (29.4–44.1)) (see Supporting Information S1: 12).

### 3.9 Risk factors

#### 3.9.1 Demographic profile and physical characteristics

Overall, there were no clear trends in the demographic profile and physical characteristics of the injured players across sex and level of play. Injured forwards from women's senior national teams, on average, were 0.9 years older (*p* = 0.008), and injured backs tended to be 0.7 cm taller (*p* = 0.057) than all forwards and backs, respectively (Fuller et al., 2021). In men's senior local level 1 teams, backs were 4.7 years younger than forwards (*p* = 0.02) and backs sustained injuries at a higher rate than forwards (risk ratio (RR) = 1.7 [0.9–3.2]; *p* = 0.08) (Lopez et al., 2012). However, injured players from men's senior national teams also tended to be older but lighter, yet this was not statistically significant (Fuller et al., 2015a). There were also no differences in age between injured and uninjured men's senior regional players (Cruz‐Ferreira et al., 2018). In men's senior regional level 2, players were taller (*p* = 0.01), heavier (*p* < 0.001) and less experienced (*p* = 0.007) than players from regional level 3 teams, and these level 3 players had a higher injury incidence rate than level 2 players (197/1000 player‐hours (91.5–374.2) versus 118/1000 player‐hours (73.1–180.9)) (Cruz‐Ferreira et al., 2018).

#### 3.9.2 Injury timing in matches, tournaments and the season

Findings consistently showed that there were increased injury proportions and/or injury incidence rates later on in matches, tournaments, and the season. In contrast, more severe injuries occurred sooner during tournaments and the season. Sixty percent (54.4–65.6) of all injuries occurred in the second half of matches, which was significantly greater than the proportion of injuries sustained in the first half (40.0% (34.4–45.6)) (see Supporting Information S1: 13). In men's senior national level 1 teams, players sustained more injuries on the second day of the tournaments as compared to the first (269 vs. 541 injuries). The proportion of second‐half injuries also increased from the first to the last match of any given day and from the first to the last day of the tournaments (Day 1: RR = 0.6–2.1 vs. Day 2: RR = 1.5–3.0) (Fuller et al., 2016). In men's senior national level 2 and 3 tournaments, where the number of matches played fluctuated from year to year, there was a significantly higher injury incidence rate during tournaments where more matches were played (Xu et al., 2021). During these tournaments, the injury incidence rates on the third day were consistently greater than on the first day, however, not always greater than on the second day (Xu et al., 2021). In addition, a higher concussion incidence rate (RR = 2.4 (1.1–5.0); *p* = 0.022) but not severity (*p* = 0.055) was seen in the second of the two consecutive paired tournaments in men's senior national teams (Fuller et al., 2015a). Likewise, the timing of injuries within a match also played a role in men's senior national teams, where 91.2% (81.6–100.0) of all concussions were sustained by players who started the match (Fuller et al., 2015a). In contrast, regional and local teams' concussion incidence rates were highest in the first three matches of tournaments (second match: 47.4%; third match: 22.8%; first match: 21.1% and *p* = 0.041) (Lopez et al., 2016). An earlier study investigating men's senior national level 1 teams also showed that there was no significant difference in the injury incidence rate from the first tournament as compared to the next consecutive follow‐on paired tournament in men's senior national teams (*p* = 0.490) (Fuller et al., 2015b). These authors also found no significant effect of distances travelled by teams before the beginning of the tournaments on injury risk (Fuller et al., 2015b).

When considering injury severity in relation to the time course of the season, a significantly shorter survival time to the onset of severe injuries was noted in senior local teams (>28 days absent from play) in comparison to slight and moderate injuries (<28 days absent from play) (24.8 vs. 70.5 h; *X* ^2^ = 30.3 and *p* = < 0.001). Therefore, more severe injuries occurred earlier in the season (Rizi et al., 2017). In women's regional and local teams, 93% of all severe injuries also occurred within the first three matches of tournaments (Ma et al., 2016). However, concussions sustained by men and women in the last three matches of tournaments accounted for 23.8 more days absent from play (*p* = 0.049) as compared to the first half of the tournament (Lopez et al., 2016). Likewise, statistically significantly more men's senior national players who sustained concussions in the first of the paired tournaments returned to play sooner than players who sustained concussions in the second of the paired tournaments (8.3% in the second tournament vs. 50.0% in the first tournament returned to play ≤7 days; *p* = 0.003) (Fuller et al., 2015a).

#### 3.9.3 Fitness

Only two studies reported on fitness and conditioning data in relation to injury risk (Cruz‐Ferreira et al., 2018; Rizi et al., 2017). The first study's multivariate regression analysis showed that gender, speed, agility and hip flexor flexibility were able to significantly predict the time to onset of a time‐loss injury in men's senior local level 3 players. Slower and less agile players also had a 3.5 and 2 times greater chance of sustaining a severe time‐loss injury, respectively (Rizi et al., 2017). The second study dealing with men's senior regional level 3 teams showed that injured players participated in weight training less often (−1.47 h; *p* = 0.048) and had a lower overall training load (12.1 h; *p* = 0.021) as compared to uninjured players (Cruz‐Ferreira et al., 2018). However, injured players from level 2 teams from the same study had a higher training load than uninjured players (*p* = 0.521), especially in players who trained rugby‐7s and rugby‐15s concurrently, compared to those who did not (RR = 3.2 (1.4–7.4); *p* = 0.011) (Cruz‐Ferreira et al., 2018).

#### 3.9.4 Previous injuries

The presence of a previous injury consistently increased the risk of a subsequent injury. However, its influence on injury severity differed when discriminating between all injuries and concussions. In Australian senior national level 1, 95.2% of players sustained a new subsequent injury over a period of two seasons. These data also showed that the number of days injury‐free gradually reduced between each subsequent new injury (Toohey et al., 2019). However, 80.7% of subsequent injuries were different diagnoses from the initial injury (Toohey et al., 2019). Players also tended to play fewer minutes, cover less overall distance and sprinting distance before the next subsequent injury (Toohey et al., 2019). Also, subsequent injuries tended to decline in time‐loss and were classified in lower injury severity categories (Toohey et al., 2019). In the USA regional and local players, 43.2% of re‐injuries were concussive injuries (Lopez et al., 2016). Players who sustained multiple concussions within the season were more severely injured than players who only sustained one new concussive injury (13.6 more days absent; *p* = 0.030) (Lopez et al., 2016).

#### 3.9.5 Playing conditions

In an annual tournament played in Singapore, there was a trend that higher recorded temperatures and lower mean wind speeds were linked to an increased injury incidence rate; however, this was not statistically significant (Xu et al., 2021). In the USA, where rugby‐7s is played on both natural and artificial grass, there were no differences in injury incidence rates as a function of the playing surfaces (Lopez et al., 2012; Ma et al., 2016).

#### 3.9.6 Protective equipment

The overall findings suggest that there were no significant injury‐preventative effects of protective equipment used in rugby‐7s. Mouthguard use was reported as 81.0% in women's and 71.8% in men's USA regional sevens (Lopez et al., 2016). In this same cohort, there was no significant difference between sexes in relation to incidence rates or severity of concussion sustained as a function of mouthguard use (*p* = 0.752) (Lopez et al., 2016). Players wearing scrum caps sustained injuries with greater mean severity than players who wore no scrum caps (51.4 vs. 28.8 days; *p* = 0.075). Furthermore, scrum cap use did not reduce injury incidence rate either (RR = 1.99 and *p* = 0.067) (Lopez et al., 2016). Similar trends with regard to the use of protective gear and injury were reported in other USA cohorts (Lopez et al., 2012; Ma et al., 2016).

## 4 DISCUSSION

This review aimed to systematically report and meta‐analyse the latest injury data available for rugby‐7s. It is the first comprehensive rugby‐7s review to include injury data for both women and men, all levels of play, match and training injury data, along with associated risk factors (see Supporting Information S1: 14). This review represents the largest analysis of injury data in this format of the sport, accounting for a total of 5137 match injuries between 2008 and 2020. The overall pooled injury incidence rate for men was 108.99/1000 player‐hours (95% CI: 82.9–135.1; *I* ^2^ = 97.6% and *p* < 0.01) and 83.1/1000 player‐hours (45.1–121.2; *I* ^2^ = 96.8% and *p* < 0.01) for women. The pooled injury severity for men was 36.3 days (25.8–46.9; *I* ^2^ = 95.9% and *p* < 0.01) and 44.1 days (28.6–59.5; *I* ^2^ = 86.2% and *p* < 0.01) for women. These findings are equivalent to those reported in the previous systematic review (Cruz‐Ferreira et al., 2017). Based on the conservative assessment of overlapping CIs (Sainani, 2011; Schenker et al., 2001), there were no significant differences between sexes with regard to injury incidence rates and severity. However, there was a difference in mean severity across the level of play for the men's teams (*Senior Men's National Level 1* = 36.3 days (25.8–46.9) versus *Senior Men's Regional Level 3* = 8.6 days (6.2–11.0)) as well as a difference between countries from which data were collected (*Senior Men's National Australian teams* = 45.0/1000 player‐hours (38.8–51.2) versus *Pooled Estimated Senior National Men's Match injury incidence* = 105.7/1000 player‐hours (82.1–129.4)). Based on the pooled meta‐analysed proportions, adolescent and women cohorts sustained the greatest proportions of concussion (Lopez et al., 2020; Nabhan et al., 2016) and experienced the greatest injury burden (Lopez et al., 2016, 2020). Proportions based on the meta‐analyses also demonstrated that significantly more match injuries occurred in the second half (60.3% (55.0–65.5)), were acute (92.6% (91.2–94.1)), located at the lower limb (55.4% (49.7–61.1)), diagnosed as joint/ligament (41.9% (37.6–46.2)), and associated with contact (78.4% (74.1–82.6)) through the mechanism of being tackled (33.8% (29.5–38.1)) (except for tackling = 24.5% (17.5–31.6)). This review found that injury incidence rates and severity were influenced by the timing of injuries during matches, tournaments and the season. Furthermore, when injuries occurred also varied by levels of play and type of injury (Fuller et al., 2015a, 2016; Lopez et al., 2016; Ma et al., 2016; Rizi et al., 2017; Xu et al., 2021). This review also showed that lower levels of physical conditioning (Rizi et al., 2017), time spent training (Cruz‐Ferreira et al., 2018), and the presence of a previous injury (Lopez et al., 2016; Toohey et al., 2019) may all be associated with injury risk in rugby‐7s. Four of the included studies were of lower methodological quality than the other 16 as they did not report how missing data or follow‐ups were handled; conduct and report appropriate statistical analyses; perform adequate stratified analyses of their cohorts; describe the subjects and setting in enough detail; and study an adequate sample size. These four studies investigated adolescent, local level 2, regional level 2, and national level 2 and 3 teams. The findings from this review satisfy step 1 and step 2 of the TRIPP model, setting the scene for the development of injury prevention strategies across the domains of equipment, training strategies and policy change in rugby‐7s.

### 4.1 No significant differences between women's and men's injury rates, types, mechanisms and risk factors

There were no statistically significant differences between women's and men's injury outcomes. This may have been influenced by the fact that men's teams had more exposure than women's teams at every comparable level of play (women's vs. men's: 655.2 h vs. 1253.9 h (Fuller et al., 2017); 511.7 h versus 1394.2 h (Fuller et al., 2017); and 454 versus 1646 h (Lopez et al., 2020)) except at the senior Rio Olympics where the number of teams and matches were fixed. There were also more men's than women's studies available in the rugby‐7s literature. Men's teams have been studied from as early as 2008, whereas studies tracking women's rugby‐7s were only published from 2013 onwards. This may be due to the professionalism of women's rugby‐7s growing at a slower rate as compared to the men's game. Only recently (2023/2024 season) has women's rugby‐7s progressed to having an equal number of competitive teams that participate in an equal number of tournaments to men's rugby‐7. With that said, our findings should be considered along with other studies that have shown differences in the technical and physical demands between women and men in team‐based collision sports (Dane et al., 2022). Dane et al. (2022) notes that preparatory strategies for men's sports will not effectively translate over to women's sports. Likewise, more women‐specific studies are needed across all levels of play in rugby‐7s to draw more substantial conclusions from these injury data.

### 4.2 Women and adolescent players suffer the greatest burden from concussions

In comparison to senior cohorts, adolescent teams (girls and boys) playing in the USA (Lopez et al., 2020) and USA adolescent teams (girls and boys) playing in the 2014 Youth Olympics (Nabhan et al., 2016), sustained the greatest proportions of concussions. USA adolescent teams (girls and boys) (Lopez et al., 2020) and USA senior women's regional and local players (Lopez et al., 2016) experienced the greatest injury burden as a consequence of concussion. Despite adolescent concussion only being studied in the USA—in comparison to senior teams—these findings may suggest that adolescent rugby‐7s players (girls and boys) are at high‐risk of concussion and the consequences thereof. Gardener et al. described younger players as having less musculature, experience and expertise with regard to tackling technique as compared to their older counterparts (Gardner et al., 2014), which may contribute to their increased concussion risk. In a review of rugby union injury epidemiology, these anthropometric and physiological differences have also been noted between men and women (West et al., 2021). In a rugby union and rugby league scoping review, den Hollander, Ponce, et al. (2021) identified tackle and ball‐carrying techniques—that when performed—are associated with reduced injury rates. Specifically in adolescent studies, non‐concussive tackle events had higher technical scores for “head placement on the correct side of ball‐carrier”, “shoulder usage”, and “leg drive into contact” (Hendricks et al., 2015). Thus, anthropometric, physiological, and/or technical outcomes may be the risk factors linked to the high concussion rates in these cohorts. However, as has been shown in adolescent rugby‐15s (West et al., 2021; Shill et al., 2022; Barden, Quarrie, et al., 2021), more injury surveillance and injury mechanism research are needed to identify specific predisposing factors for concussion in rugby‐7s.

### 4.3 The rugby‐7s injury risk profile

The injury risk profile of rugby‐7s players seemed to be unaffected by the use of protective equipment, playing conditions, playing surfaces, and player demographics. Factors that may alter the injury risk profile of rugby‐7s players include the timing of injuries in matches, tournaments, and the season, player fitness, and the presence of a previous injury. Preliminary evidence suggests that increased levels of fatigue may increase the injury incidence rate in rugby‐7s. More injuries occur during the last days of tournaments (Fuller et al., 2016), and in the second halves, where the proportion of second‐half injuries increased from the first to the last matches of tournaments (Fuller et al., 2016). Furthermore, tournaments with a greater number of matches were associated with a higher injury incidence rate (Xu et al., 2021). The increased cumulative fatigue during these tournaments (compared to tournaments with fewer matches) may explain the association with higher injury rates. On the other hand, a lack of fatigue was linked to a greater injury severity. The mean time to onset of severe injury was just longer than the first 24 h of rugby played within a season. Ma et al. (2016) also showed that 93% of all severe injuries occurred within the first three matches for women's players. This was likely the consequence of players' increased capacity for speed, power, and strength during high‐impact collisions and tackles earlier during the season, tournaments, and matches. An alternative hypothesis is that during the early stages of the season, players are still adjusting and adapting to the demands of the game.

For concussions, injury severity seemed to increase with greater cumulative fatigue in senior men's cohorts. Concussions sustained in the last three matches of a tournament (Lopez et al., 2016) and the second of two‐paired tournaments accounted for more time‐loss than injuries sustained sooner during tournaments and seasons (Fuller et al., 2015a). This may be a result of an accelerated return‐to‐play for players who sustained concussions in the first part of the paired tournaments. This may be in an attempt to maintain their best travelling squad's availability to play in the next consequent tournament. Concussions in the second part of the paired tournaments may have been dealt with more conservatively, as the following tournament was likely only in a month. Apart from the severity, concussion incidence rates also seemed to increase later in national tournaments (Fuller et al., 2015b). Furthermore, national players who played more (minutes in a match) also sustained concussions at a higher rate (Fuller et al., 2015a). This may be evidence that an increase in fatigue plays a role in the increased incidence rate. An increase in fatigue has been associated with a reduction in stability and muscle stiffness in the trunk and spine (Granata et al., 2004; Van Dieën et al., 2012). Therefore, increased fatigue and a consequent lack of trunk and spinal control may be a plausible mechanistic factor in the rugby‐7s injury risk profile. Conversely, in regional and local players, the greatest proportion of concussive injuries occurred in the first three matches of a tournament (Lopez et al., 2016). In this case, the increased running speeds and explosive force production may be linked to the lowered fatigue state during these matches of the tournament. This may lead to higher impact tackles and thus may be a plausible explanation for the increased concussion incidence for these levels of play. Therefore, since changes in fatigue levels seem to play a role in shaping the injury profile of rugby‐7s players, more research is needed that directly measures markers of fatigue and its association with rugby‐7s injuries. This is especially important due to the unique nature of the elite SWS schedule and the limited rest and recovery times between matches and tournaments.

Only one study discussed the link between players' fitness and their injury risk (Rizi et al., 2017). Slower, less agile, less flexible players, had an increased risk of severe injury (Rizi et al., 2017). As with fatigue, training load and the presence of a previous injury both increased and decreased the injury risk. Players at higher levels of play, with greater training loads, had a greater injury RR (Cruz‐Ferreira et al., 2018). Whereas, players at lower levels of play, with fewer hours of rugby and weight training, had an increased injury RR (Cruz‐Ferreira et al., 2018). The presence of a previous injury not only increased the risk of another injury but also reduced the time in which the new injury is sustained (Toohey et al., 2019). For concussive injuries, sustaining multiple concussions meant that these consequent injuries were more severe and accounted for more time‐loss.

### 4.4 Implications for researchers, practitioners and coaches

This review supports the notion that rugby‐7s has one of the highest injury incidences across all rugby codes and sports (108.5/1000 player‐hours vs. 89.1–96.0 injuries/1000 player‐hours in rugby‐15s and 87.7 injuries/1000 player‐hours in rugby league (Cruz‐Ferreira et al., 2017)). With that said, men's rugby‐15s have comparable injury profiles to rugby‐7s with an overall injury incidence rate of 91/1000 player‐hours (77–106); overall, injury severity of 27 days (Cruz‐Ferreira et al., 2018; Fuller et al., 2017; Lopez et al., 2012, 2016, 2020; Ma et al., 2016; Reis et al., 2018; Rizi et al., 2017; Schenker et al., 2001; Toohey et al., 2019), 48.6% of injuries occurring at the lower limb and 45.8% of injuries occurring during the tackle, with no statistical difference between injury incidence rates between forwards and backs (*p* = 0.950). These findings may suggest that rugby‐15s injury prevention strategies may sufficiently translate over to rugby‐7s due to their comparable injury profiles. However, such a conclusion may be unwise as it ignores the physical, technical and performance characteristics between the two sports.

Moreover, the results of this review may imply that a single rugby‐7s‐specific injury prevention strategy may be effective for both men and women. Such an interpretation would be misguided, as one cannot ignore the inherent differences between the men's and women's games. Dane et al. (2023) recently described how rugby tackle coaching should be re‐imagined for women's players. The aim should be to break down the embedded cultural and gendered factors that currently constrain the teaching, training and performance of the tackle in the women's game. Dane et al. (2022) also explained that preparatory strategies for men's collision sports do not successfully translate over to women's players. Arguably, injury prevention should be dealt with similarly. Therefore, we should aim to purposefully create sex‐specific strategies to protect rugby‐7s players from injury. However, considering this area is still in its infancy, effective rugby‐15s injury prevention strategies could be used for both men and women rugby‐7s players until more specific strategies are developed (Barden, Bekker, et al., 2021).

In contrast to rugby‐7s, both rugby‐15s and rugby league research have already used video analysis studies to shed light on specific match determinants and technical tackle profiles associated with injury risk (den Hollander, Ponce, et al., 2021; den Hollander et al., 2018). Thus, they have been able to develop and implement effective injury prevention strategies linked to the rugby tackle (Barden, Bekker, et al., 2021). Therefore, injury mechanism video analysis studies are needed to identify technical and match determinants that may be associated with rugby‐7s injuries.

## 5 LIMITATIONS

This review employed conservative statistical analysis. Even though this may seem like a limitation, it has been suggested that this form of statistical analysis is more clinically applicable to sports science and sports medicine (Sainani, 2011). This review also did not exclude studies based on their respective quality assessments but only based on the inclusion and exclusion criteria (see Supporting Information S1: 15). However, this may be construed as a limitation, since this meta‐analysis was the first of its kind, all studies were included. The studies that scored lower on the quality assessment were conducted mainly on adolescent teams and teams participating at lower levels of play. Considering this, future research on these cohorts needs to ensure proper data collection, reporting, and analyses. Even though ‘concussive injury’ was not included as a search term, concussive injury data were still extracted. Therefore, it must be noted that specific concussion injury studies may have been missed in the search parameters. It is also noteworthy that most senior men's studies following the SWS were longitudinal cohorts that were continually updated and published. Therefore, including all available literature meant that there may be overlapping data between studies within this review. With regards to the limitations of the studies included, there were too few studies reporting women's and adolescent's data as well as too few studies reporting risk factors and mechanisms associated with these injury profiles. Even though all studies consistently reported injury location in four main categories (head/neck, upper limb, trunk, and lower limb), there were inconsistencies in the use of sub‐category classifications. This was mainly found in the classification of: head and face; shoulder and clavicle; wrist, fingers, and hands; pelvis and buttock; hip and groin; calf, shin, and lower leg; and foot and toes. With regards to the classification of injury types, there was also no consistent use of either the rugby union consensus statement (Fuller et al., 2007) or the IOC consensus statement (Bahr et al., 2020). All rugby‐specific studies should follow the rugby union consensus statement on injury definitions and data‐capturing procedures epidemiology and reporting (Fuller et al., 2007).

## 6 CONCLUSION

This systematic review has included all available injury epidemiology data for rugby‐7s and confirmed that rugby‐7s accounts for one of the highest injury incidence rates and severities across all sports. Pooled estimated proportions based on meta‐analyses showed that significantly more match injuries occurred in the second half, were acute, located at the lower limb, diagnosed as joint/ligament injuries, and caused by contact through the mechanism of being tackled. Overall, the rugby‐7s injury risk and/or injury incidence rates increased due to factors such as transient fatigue, player fitness, and the presence of previous injuries. Player demographics and physical characteristics, use of protective equipment and playing conditions did not change the injury risk profile in rugby‐7s. There were no statistically significant differences between women's and men's injury incidence rates, severity, types, mechanisms, and risk factors. However, due to the influence of gendered and cultural factors, one cannot ignore the inherent differences between the women's and men's games. The development of injury prevention strategies should thus still aim to be sex‐specific. The findings from this review will help pave the way forward beyond the foundational stages of injury prevention research in rugby‐7s. It will form the basis from which future injury prevention strategies across the domains of equipment, training strategies, and policy change can be developed.

## AUTHOR CONTRIBUTION

Sharief Hendricks and Muhammed Rizaan Behardien conceptualised the research project, study design, and registered the protocol on OSF Registries. Muhammed Rizaan Behardien completed the full literature search, retrieval, critical appraisal, data extraction, data synthesis, and data analysis and complied with the initial manuscript draft. Janesh Ganda acted as the second reviewer. Kathryn Dane completed data extraction for 10% of the included studies. All authors have provided input on the process and write‐up of the final manuscript.

## CONFLICT OF INTEREST STATEMENT

SH is the social media editor for the European Journal of Sport Science and an Associate Editor.

## PATIENT AND PUBLIC INVOLVEMENT

This systematic review and meta‐analyses did not include patient or public involvement in the development of the research questions or methodologies due to the nature of the research.

## Supporting information

Supporting Information S1

## Data Availability

All data relevant to the study are included in this review or uploaded as supplementary information. The information can also be publicly accessed by using the references provided for the 20 included studies in this review.
