# Supplementary material for: Paving the path for injury prevention in rugby‐7s: A systematic review and meta‐analysis
Source: Eur J Sport Sci. 2024 Jun 27;24(9):1209–27. doi: 10.1002/ejsc.12156 (PMC11369338; doi:10.1002/ejsc.12156)
Supplement: Supplementary file 1 — Supporting Information S1 [file EJSC-24-1209-s001.docx]

**Electronic Supplementary Material**

**Paving the path for injury prevention in rugby-7s: A systematic review and meta-analysis.**

Muhammed Rizaan Behardien^1,2^, Janesh Ganda^3,4^, Kathryn Dane^5^, Stephen West^6,7,8^, Carolyn Emery^8,9,10,11,12,13,14^, Ben Jones^1,15,16,17^, Sharief Hendricks^1,2,15.18^

*^1^UCT Research Centre for Health through Physical Activity (HPALS), Lifestyle and Sport, Division of Physiological Sciences, Department of Human Biology, Faculty of Health Sciences, University of Cape Town, Cape Town, South Africa*

*^2^Division of Physiological Sciences, Department of Human Biology, Faculty of Health Sciences, University of Cape Town, South Africa*

*^3^Sports Rehab Centre, Cape Town, South Africa*

*^4^Wits Sport and Health (WiSH), School of Clinical Medicine, Faculty of Health Sciences, University of the Witwatersrand, Johannesburg-Braamfontein, South Africa*

*^5^Discipline of Physiotherapy, School of Medicine, Trinity College Dublin, Ireland*

*^6^Centre for Health, and Injury & Illness Prevention in Sport, Department for Health, University of Bath*

*^7^UK Collaborating Centre on Injury and Illness Prevention in Sport (UKCCIIS), University of Bath*

*^8^Sport Injury Prevention Research Centre, Faculty of Kinesiology, University of Calgary*

*^9^O'Brien Institute of Public Health, University of Calgary, Calgary, Canada*

*^10^Hotchkiss Brain Institute, University of Calgary, Calgary, Canada*

*^11^Alberta Children's Hospital Research Institute, University of Calgary, Calgary, Canada*

*^12^McCaig Institute for Bone and Joint Health, University of Calgary, Calgary, Canada*

*^13^Department of Community Health Sciences, Cumming School of Medicine, University of Calgary, Calgary, Canada*

*^14^Department of Pediatrics, Cumming School of Medicine, University of Calgary, Calgary, Canada*

*^15^Carnegie Applied Rugby Research (CARR) centre, Carnegie School of Sport, Leeds Beckett University, Leeds, UK*

*^16^Premiership Rugby, London, UK*

*^17^England Performance Unit, Rugby Football League, UK*

*^18^International Federation of Sports Medicine (FIMS) Collaborative Centre of Sports Medicine, University of Cape Town, Cape Town, South Africa*

Corresponding Author: Dr Sharief Hendricks - [Sharief.hendricks@uct.ac.za](mailto:Sharief.hendricks@uct.ac.za)

**Supplementary Material:**


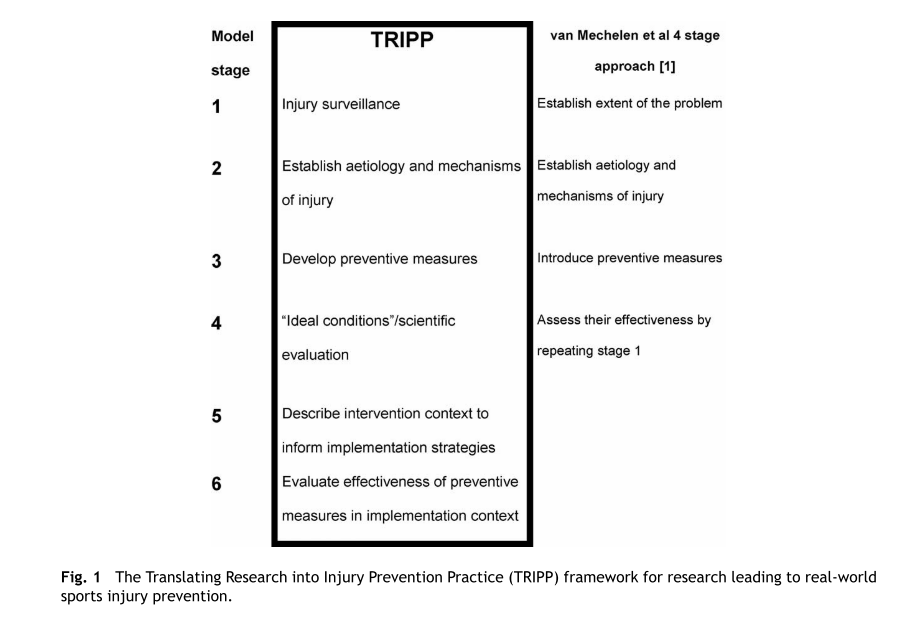
**SUPPLEMENTARY MATERIAL 1**

Figure 1: The Translating Research into Injury Prevention Practice (TRIPP) framework.

*Finch C. A new framework for research leading to sports injury prevention. J Sci Med Sport. 2006;9:3–9.*

**SUPPLEMENTARY MATERIAL 2**

Full search details:

SCOPUS (n=317):

( ( "rugby" AND ( {sevens} OR {7s} OR {rugby-7s} OR {rugby7s} OR {rugby 7s} OR {world series} OR "tackle collision" OR {collision sport} ) ) ) AND ( ( "injur*" OR "athlet*" OR "athletic Injur*" OR "mechan*" OR risk OR factor ) OR ( "epidemiol*" OR incidence OR rates ) )

Limits = final publication stage, only reviews or article document types, and only found in journals

Web of Science (n=421):

(((rugby AND ("sevens" OR "7s" OR "rugby-7s" OR "rugby7s" OR "rugby 7s" OR "sevens world series")) OR “tackle collision” OR “collision sport”) AND ((“injur*” OR “athlet*” OR “athletic Injur*” OR “mechan*” OR risk OR factor) OR (epidemiol* OR incidence OR rates)))

Limits: none

Ebsohost (n=420):

((rugby AND (“sevens” OR “7s” OR “rugby-7s” OR “rugby7s” OR “rugby 7s” OR “sevens world series” OR “tackle collision” OR “collision sport”))) AND ((“injur*” OR “athlet*” OR “athletic injur*” OR “mechan*” OR risk OR factor) OR (epidemiol* OR incidence OR rates))

Limits: none

Pubmed (n=374):

((rugby AND (“sevens” OR “7s” OR “rugby-7s” OR “rugby7s” OR “rugby 7s” OR “sevens world series” OR “tackle collision” OR “collision sport”))) AND ((“injur*” OR “athlet*” OR “athletic injur*” OR “mechan*” OR risk OR factor) OR (epidemiol* OR incidence OR rates))

Limits: none

All databases were last search on 20 March 2024.

**SUPPLEMENTARY MATERIAL 3**

Table 1: Assessment of methodological quality using the ‘JBI Critical Appraisal Checklist of Studies Reporting Prevalence Data’

| **Studies** | **Questions within the checklist** | | | | | | | | | |
| --- | --- | --- | --- | --- | --- | --- | --- | --- | --- | --- |
|  | 1 | 2 | 3 | 4 | 5 | 6 | 7 | 8 | 9 | Total |
| Fuller, Taylor & Molloy, 2010 | Yes | Yes | Yes | Yes | Yes | Yes | Yes | Yes | Yes | 9 |
| Lopez et al., 2012 | Yes | Yes | Yes | No | Yes | Yes | Yes | Yes | Yes | 8 |
| Fuller, Taylor & Raftery, 2015a | Yes | Yes | Yes | Yes | Yes | Yes | Yes | Yes | Yes | 9 |
| Fuller, Taylor & Raftery, 2015b | Yes | Yes | Yes | Yes | Yes | Yes | Yes | Yes | Yes | 9 |
| Lopez et al., 2016a | Yes | Yes | Yes | Yes | Yes | Yes | Yes | Yes | Yes | 9 |
| Ma et al., 2016 | Yes | Yes | Yes | Yes | Yes | Yes | Yes | Yes | Yes | 9 |
| Nabhan et al., 2016 | Yes | Yes | No | No | No | Yes | Yes | No | Unclear | 4 |
| Fuller, Taylor & Raftery, 2016 | Yes | Yes | Yes | Yes | Yes | Yes | Yes | Yes | Yes | 9 |
| Fuller, Taylor & Raftery, 2017 | Yes | Yes | Yes | Yes | Yes | Yes | Yes | Yes | Yes | 9 |
| Rizi et al., 2017 | Yes | Yes | No | Yes | No | Yes | Yes | No | Unclear | 5 |
| Soligard et al., 2017 | Yes | Yes | Yes | Yes | Yes | Yes | Yes | Yes | Unclear | 8 |
| Cruz-Ferreira et al., 2018 | Yes | Yes | Yes | Yes | No | Yes | Yes | Yes | Unclear | 7 |
| Fuller, 2018a | Yes | Yes | Yes | No | Yes | Yes | Yes | Yes | N/A | 8 |
| Reis, Gordo & Marques, 2018 | Yes | No | No | Yes | Yes | Yes | Unclear | No | No | 4 |
| Toohey et al., 2019 | Yes | Yes | Yes | No | Yes | Yes | Yes | Yes | Yes | 8 |
| Fuller & Taylor, 2020 | Yes | Yes | Yes | Yes | Yes | Yes | Yes | Yes | Yes | 9 |
| Lopez et al., 2020 | Yes | Yes | Yes | Yes | Yes | Yes | Yes | Yes | Yes | 9 |
| Steffen et al., 2020 | Yes | Yes | Yes | Yes | Yes | Yes | Yes | Yes | Yes | 9 |
| Fuller & Taylor, 2021 | Yes | Yes | Yes | Yes | Yes | Yes | Yes | Yes | Yes | 9 |
| Xu et al., 2021 | Yes | Yes | Yes | No | No | Yes | Yes | No | No | 5 |

Note: The tool includes the following questions: 1. Was the sample frame appropriate to address the target population?, 2. Were study participants sampled in an appropriate way?, 3. Was the sample size adequate?, 4. Were the study subjects and the setting described in detail?, 5. Was the data analysis conducted with sufficient coverage of the identified sample?, 6. Were valid methods used for the identification of the condition?, 7. Was the condition measured in a standard, reliable way for all participants?, 8. Was there appropriate statistical analysis?, 9.Was the response rate adequate, and if not, was the low response rate managed appropriately? Available at: <https://jbi.global/critical-appraisal-tools>.

**SUPPLEMENTARY MATERIAL 4**

Table 2: Training data extracted from selected studies.

|  | **Reference** | **Setting** | **Level of play** | | | **Injury definition** | **Surveillance period** | **Sample** | **Sample injured**  **(proportion, %)** | **Injury count** | **Total exposure (hrs)** | **Overall incidence**  **(inj/1000ph, 95% CI)** | **Time loss injuries**  **(proportion, %)** | **Medical attention**  **injuries (proportion, %)** | **Mean severity**  **(days, 95% CI)** | **Injury burden** |
| --- | --- | --- | --- | --- | --- | --- | --- | --- | --- | --- | --- | --- | --- | --- | --- | --- |
| **Senior, National Teams** | | | |  |  |  |  |  |  |  |  |  |  |  |  |  |
|  | Soligaard et al. (Soligard et al., 2017) | 2016 Rio Olympics - All Teams | National, Level 1 + 2 | | | Junge et al., 2008 | Rio 2016 | Overall = 291 | 8 (2,75%) | 8 | N/R | N/R | N/R | N/R | N/R | N/R |
|  | Fuller, Taylor & Raftery (Fuller, Taylor & Raftery, 2017) | HSBC SWS - All Teams | National, Level 1 | | | Fuller, 2007 | 2014-2015 | Women = 197 | N/R | 6 | 77,4 ^F^ | 0,95  (0,4-2,1) | 100% | N/A | 27 | 25,7 |
|  |  |  |  |  |  |  |  | Men = 331 | N/R | 11 | 73,0 ^F^ | 0,8  (0,4-1,5) | 100% | N/A | 31,5 | 25,2 |
|  |  | HSBC SWS - All Teams | National, Level 1 | | | Fuller, 2007 | 2015-2016 | Women = 221 | N/R | 10 | 78,1 ^F^ | 2,2  (1,2-4,1) | 100% | N/A | 67,1 | 147,6 |
|  |  |  |  |  |  |  |  | Men = 340 | N/R | 14 | 73,7 ^F^ | 1,2  (0,7-2) | 100% | N/A | 39,5 | 47,4 |
|  |  | Rio Olympics 2016 - All Teams | National, Level 1 + 2 | | | Fuller, 2007 | Rio 2016 | Women = 148 | N/R | 1 | 73,2 ^F^ | 1,1 | 100% | N/A | N/R | N/A |
|  |  |  |  |  |  |  |  | Men = 152 | N/R | 0 | 86,8 ^F^ | 0 | 100% | N/A | 0 | N/A |
|  | Fuller (Fuller, 2018) | HSBC SWS - All Teams | National, Level 1 | | | Fuller, 2007 | 2008-2016 | Men | N/R | 34 | 37389,0 | 0,9  (0,7-1,2) | 100% | N/A | 43,5  (31,1-5,9) | 39,6 |
| **Senior, Local Teams** | | | | |  |  |  |  |  |  |  |  |  |  |  |  |
|  | Rizi et al. (Rizi et al., 2017) | University Rugby - Hong Kong | Local, Level 2 | | | Fuller, 2008 | 2014-2015 | Overall = 104 | NR | 15 | 4525,0 | 3,3 | 100% | N/A | 8,62  (SD +/- 8) | 28,5 |

*Note: N/A = not applicable to the study. N/R = not reported within the study*

**SUPPLEMENTARY MATERIAL 5**


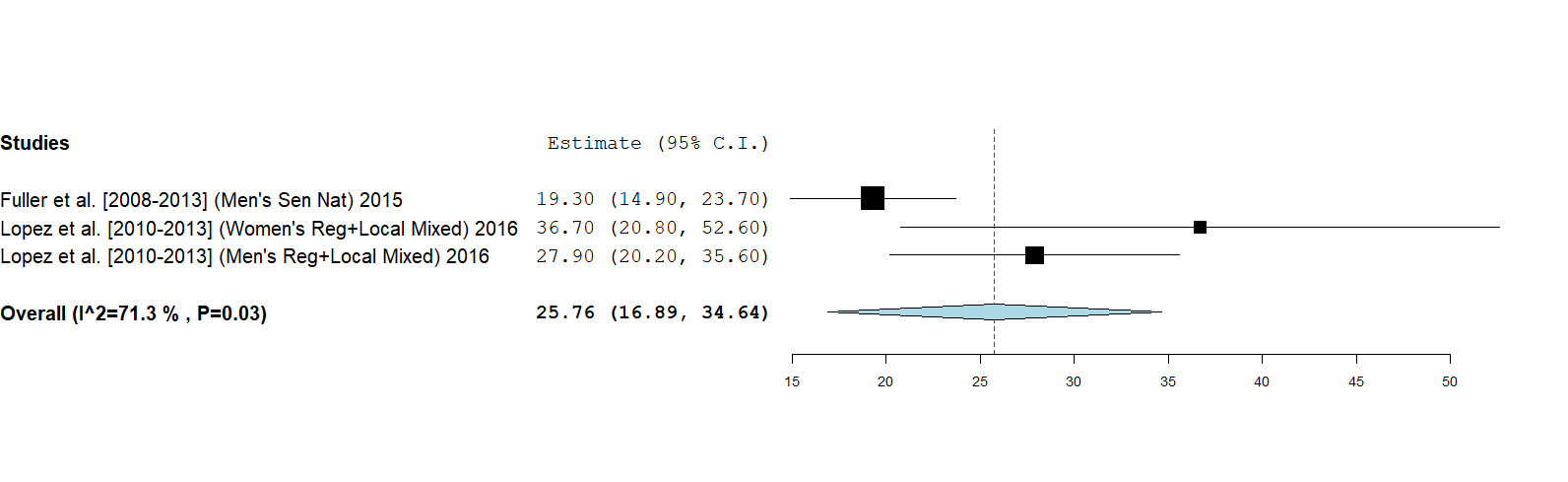
**Figure 2:** Severity of concussions by level of play and sex.

**SUPPLEMENTARY MATERIAL 6**

**Figure 3:** Injury burden plot by type of injury

*Note: Male muscle/tendon injuries were left out of the analysis as it was reported incorrectly.*

**SUPPLEMENTARY MATERIAL 7**

Table 3: Match injuries as a function of location. The meta-analysis combined all proportions from the respective studies.

| **Location** | **Sample Classification** | **Number of samples** | **Specific injury count** | **Meta-analysed proportion (95% CI)** |
| --- | --- | --- | --- | --- |
| Lower Limb | All Samples | 22 | 2519 | 55.4% (49.7-61.1) |
|  | Senior National Women | 5 | 362 | 52.5% (42.8-62.1) |
|  | Senior National Men | 9 | 1980 | 57.5% (51.4-63.5) |
|  | Senior Regional Men | 2 | 25 | 55.6% ((-)121.0-232.2) |
|  | Senior Local Men | 1 | 7 | 14.6% (N/R) |
|  | Youth National Overall | 1 | 42 | 68.2% (N/R) |
|  | Youth Regional Women | 1 | 7 | 38.9% (18.2–64.5) |
|  | Youth Regional Men | 1 | 21 | 41.2% (28.2–55.5) |
| Upper Limb | All Samples | 22 | 901 | 19.8% (16.5-23.1) |
|  | Senior National Women | 5 | 142 | 20.6% (15.7-25.4)­ |
|  | Senior National Men | 9 | 665 | 19.3% (14.3-24.3) |
|  | Senior Regional Men | 2 | 14 | 31.1% ((-228.1-290.3) |
|  | Senior Local Men | 1 | 15 | 31.3% (N/R) |
|  | Youth National Overall | 1 | 3 | 4.5% (N/R) |
|  | Youth Regional Women | 1 | 6 | 33.3% (14.4–59.7) |
|  | Youth Regional Men | 1 | 16 | 31.4% (19.9–45.7) |
| Head | All Samples | 21 | 808 | 17.8% (14.8-20.8) |
|  | Senior National Women | 5 | 134 | 19.4% (11.7-27.2) |
|  | Senior National Men | 9 | 587 | 17.0% (12.8-21.3) |
|  | Senior Regional Men | 1 | 4 | 14.8% (3.1–30.4) |
|  | Senior Local Men | 1 | 16 | 33.3% (N/R) |
|  | Youth National Overall | 1 | 14 | 22.7% (N/R) |
|  | Youth Regional Women | 1 | 5 | 27.8% (10.9–54.7) |
|  | Youth Regional Men | 1 | 11 | 21.6% (12.1–35.4) |
| Trunk | All Samples | 21 | 297 | 6.6% (4.5-8.6) |
|  | Senior National Women | 5 | 52 | 7.5% (2.9-12.2) |
|  | Senior National Men | 9 | 212 | 6.2% (4.0-8.3) |
|  | Senior Regional Men | 1 | 1 | 3.7% (0.0–12.9) |
|  | Senior Local Men | 1 | 9 | 18.8 (N/R) |
|  | Youth National Overall | 1 | 3 | 4.5% (N/R) |
|  | Youth Regional Women | 1 | 0 | 0.0% (0.0-0.0) |
|  | Youth Regional Men | 1 | 2 | 3.9% (0.9–15.0) |

*Note: N/R = not reported within the study.*

**SUPPLEMENTARY MATERIAL 8**

Table 4: Match injuries as a function of injury type. The meta-analysis combined all proportions from the respective studies.

| **Injury Type** | **Sample classification** | **Number of samples** | **Specific injury count** | **Meta-analysed proportion (95% CI)** |
| --- | --- | --- | --- | --- |
| Joint/Ligament Injury | All Samples | 21 | 1760 | 41.9% (37.6-46.2) |
|  | Senior National Women | 5 | 292 | 42.3% (30.4-54.2) |
|  | Senior National Men | 8 | 1340 | 43.2% (39.4-47.0) |
|  | Senior Regional Men | 2 | 22 | 48.9% ((-)22.3-120.0) |
|  | Senior Local Men | 1 | 11 | 22.9% (N/R) |
|  | Youth National Overall | 1 | 18 | 29.5% (N/R) |
|  | Youth Regional Women | 1 | 7 | 38.9% (18.2–64.5) |
|  | Youth Regional Men | 1 | 13 | 25.5% (15.1-39.6) |
| Muscle/Tendon injury | All Samples | 20 | 1034 | 25.3% (21.0-29.7) |
|  | Senior National Women | 5 | 182 | 26.4% (16.9-35.8) |
|  | Senior National Men | 8 | 785 | 25.3% (19.7-30.9) |
|  | Senior Regional Men | 2 | 17 | 37.8% ((-)8.6-84.2) |
|  | Senior Local Men | 1 | 18 | 37.5% (N/R) |
|  | Youth National Overall | 1 | 10 | 16.4% (N/R) |
|  | Youth Regional Women | 1 | 5 | 27.8% (10.9–54.7) |
|  | Youth Regional Men | 1 | 1 | 2.0% (0.3-13.4) |
| Skin | All Samples | 19 | 337 | 8.5% (3.6-13.4) |
|  | Senior National Women | 5 | 21 | 3.0% ((-)2.5-8.6) |
|  | Senior National Men | 7 | 268 | 8.9% (3.6-14.3) |
|  | Senior Regional Men | 2 | 6 | 13.3% ((-)139.8-166.4) |
|  | Senior Local Men | 1 | 7 | 14.6% (N/R) |
|  | Youth National Overall | 1 | 19 | 31.1% (N/R) |
|  | Youth Regional Women | 1 | 0 | 0.0% (-) |
|  | Youth Regional Men | 1 | 1 | 2.0% (0.3-13.4) |
| Neural (PNS/CNS) | All Samples | 18 | 503 | 12.7% (10.0-15.3) |
|  | Senior National Women | 5 | 97 | 14.1% (6.0-22.1) |
|  | Senior National Men | 8 | 388 | 12.5% (8.8-16.2) |
|  | Senior Regional Men | 2 | 3 | 6.7% ((-)4.8-18.1) |
|  | Senior Local Men | 1 | 8 | 16.7 (N/R) |
|  | Youth National Overall | 1 | 5 | 8.2% (N/R) |
|  | Youth Regional Women | 0 | 0 | N/R |
|  | Youth Regional Men | 0 | 0 | N/R |
| Bone Injury | All Samples | 20 | 381 | 9.2% (7.6-10.8) |
|  | Senior National Women | 5 | 81 | 11.7% (6.9-16.5) |
|  | Senior National Men | 8 | 263 | 8.5% (6.4-10.6) |
|  | Senior Regional Men | 1 | 1 | 5.6% (N/R) |
|  | Senior Local Men | 1 | 3 | 6.3% (N/R) |
|  | Youth National Overall | 1 | 5 | 8.2% (N/R) |
|  | Youth Regional Women | 1 | 2 | 11.1% (2.4–38.9) |
|  | Youth Regional Men | 1 | 8 | 15.7% (7.9-28.9) |

Table 4: *cont.*

| **Injury Type** | **Sample classification** | **Number of samples** | **Specific injury count** | **Meta-analysed proportion (95% CI)** |
| --- | --- | --- | --- | --- |
| Other | All Samples | 20 | 81 | 2.0% (0.9-3.1) |
|  | Senior National Women | 5 | 17 | 2.5% ((-)2.1-7.0) |
|  | Senior National Men | 8 | 54 | 1.7% (0.6-2.9) |
|  | Senior Regional Men | 2 | 1 | 2.2% ((-)21.3-25.7) |
|  | Senior Local Men | 1 | 1 | 2.1% (N/R) |
|  | Youth National Overall | 1 | 4 | 6.6% (N/R) |
|  | Youth Regional Women | 1 | 0 | 0.0% (-) |
|  | Youth Regional Men | 1 | 1 | 2.0% (0.3-13.4) |
| **Specific injury diagnosis** | **Sample classification** | **Number of samples** | **Specific injury count** | **Meta-analysed proportion (95% CI)** |
| Concussion | All Samples | 18 | 478 | 11.8% (9.0-14.6) |
|  | Senior National Women | 4 | 84 | 15.6% (9.6-21.6) |
|  | Senior National Men | 7 | 345 | 11.0% (6.7-15.4) |
|  | Senior Regional Men | 1 | 1 | 5.6% (N/R) |
|  | Senior Local Men | 1 | 7 | 14.6% (N/R) |
|  | Youth National Overall | 1 | 11 | 18.2% (N/R) |
|  | Youth Regional Women | 1 | 4 | 22.2% (7.7–49.5) |
|  | Youth Regional Men | 1 | 9 | 17.6% (9.2–31.1) |

*Note: N/R = not reported within the study; N/A = not applicable.*

**SUPPLEMENTARY MATERIAL 9**

Table 5: Match injuries as a function of type of onset. The meta-analysis combined all proportions from the respective studies.

| **Nature of onset** | **Sample classification** | **Number of samples** | **Specific injury count** | **Meta-analysed proportion (95% CI)** |
| --- | --- | --- | --- | --- |
| Acute | All Samples | 13 | 3349 | 92.6% (91.2-94.1) |
|  | Senior National Women | 4 | 503 | 93.5% (88.9-98.2) |
|  | Senior National Men | 7 | 2665 | 92.3% (90.7-93.8) |
|  | Senior Regional Men | 0 | 0 | (N/A) |
|  | Senior Local Men | 0 | 0 | (N/A) |
|  | Youth National Overall | 0 | 0 | (N/A) |
|  | Youth Regional Women | 1 | 18 | 100.0% (-) |
|  | Youth Regional Men | 1 | 51 | 100.0% (-) |
| Gradual | All Samples | 13 | 266 | 7.4% (5.6-9.1) |
|  | Senior National Women | 4 | 35 | 6.5% (1.9-11.1) |
|  | Senior National Men | 7 | 223 | 7.7% (6.2-9.3) |
|  | Senior Regional Men | 0 | 0 | (N/A) |
|  | Senior Local Men | 0 | 0 | (N/A) |
|  | Youth National Overall | 0 | 0 | (N/A) |
|  | Youth Regional Women | 1 | 0 | 0.0% (-) |
|  | Youth Regional Men | 1 | 0 | 0.0% (-) |

*Note: N/A = not applicable.*

**SUPPLEMENTARY MATERIAL 10**

Table 6: Injury event – Match injuries as a function of match event. The meta-analysis combined all proportions from the respective studies.

| **Match event** | **Sample classification** | **Number of samples** | **Specific injury count** | **Meta-analysed proportion (95% CI)** |
| --- | --- | --- | --- | --- |
| Tackled | All Samples | 11 | 1157 | 33.8% (29.5-38.1) |
|  | Senior National Women | 1 | 147 | 35.4% (30.7–40.0) |
|  | Senior National Men | 4 | 848 | 33.1% (32.1-34.2) |
|  | Senior Regional Men | 1 | 10 | 38.1% (17.6–60.0) |
|  | Senior Local Men | 0 | - | - |
|  | Youth Regional Overall | 1 | 23 | 32.7 (N/R) |
|  | Concussion – Senior National Men | 1 | 9 | 26.5% (11.6-41.3) |
|  | Concussion – Youth Regional and Local Women | 1 | 4 | 19.0% (2.2-35.8) |
|  | Concussion – Youth Regional and Local Men | 1 | 12 | 26.1% (13.4-38.8) |
| Tackling | All Samples | 11 | 841 | 24.5% (17.5-31.6) |
|  | Senior National Women | 1 | 109 | 26.3% (22.0–30.6) |
|  | Senior National Men | 4 | 573 | 22.4% (18.8-26.0) |
|  | Senior Regional Men | 1 | 5 | 19.0% (4.8–38.1) |
|  | Senior Local Men | 0 | - | - |
|  | Youth Regional Overall | 1 | 30 | 44.1% (N/R) |
|  | Concussion – Senior National Men | 1 | 15 | 44.1% (27.4-60.8) |
|  | Concussion – Youth Regional and Local Women | 1 | 9 | 42.9% (21.7-64.1) |
|  | Concussion – Youth Regional and Local Men | 1 | 17 | 37.0% (23.1-51.0) |
| Running | All Samples | 12 | 551 | 15.9% (10.2-21.5) |
|  | Senior National Women | 1 | 35 | 8.4% (5.7–11.0) |
|  | Senior National Men | 4 | 450 | 17.6% (13.7-21.4) |
|  | Senior Regional Men | 1 | 0 | 0.0% (-) |
|  | Senior Local Men | 1 | 6 | 12.8% (6.0-25.0) |
|  | Youth Regional Overall | 1 | 8 | 11.6% (5.8-21.8) |
|  | Concussion – Senior National Men | 1 | 0 | 0.0% (-) |
|  | Concussion – Youth Regional and Local Women | 1 | 0 | 0.0% (-) |
|  | Concussion – Youth Regional and Local Men | 1 | 0 | 0.0% (-) |
| Collision | All Samples | 12 | 403 | 11.6% (5.0-18.3) |
|  | Senior National Women | 1 | 57 | 13.8% (10.4–17.1) |
|  | Senior National Men | 4 | 319 | 12.5% (9.5-15.4) |
|  | Senior Regional Men | 1 | 3 | 9.5% (0.0–23.5) |
|  | Senior Local Men | 1 | 0 | 0.0% (-) |
|  | Youth Regional Overall | 1 | 0 | 0.0% (-) |
|  | Concussion – Senior National Men | 1 | 8 | 23.5% (9.3-37.8) |
|  | Concussion – Youth Regional and Local Women | 1 | 7 | 33.3% (13.1-53.5) |
|  | Concussion – Youth Regional and Local Men | 1 | 9 | 19.6% (8.1-31.1) |

Table 6: *cont.*

| **Match event** | **Sample classification** | **Number of samples** | **Specific injury count** | **Meta-analysed proportion (95% CI)** |
| --- | --- | --- | --- | --- |
| Ruck | All Samples | 12 | 239 | 6.9% (4.1-9.7) |
|  | Senior National Women | 1 | 37 | 8.8% (6.1–11.6) |
|  | Senior National Men | 4 | 191 | 7.4% (1.4-13.5) |
|  | Senior Regional Men | 1 | 0 | 0.0% (-) |
|  | Senior Local Men | 1 | 2 | 4.3% (1.0-14.0) |
|  | Youth Regional Overall | 1 | 3 | 4.3% (1.4-13) |
|  | Concussion – Senior National Men | 1 | 1 | 2.9% (0-8.6) |
|  | Concussion – Youth Regional and Local Women | 1 | 0 | 0.0% (-) |
|  | Concussion – Youth Regional and Local Men | 1 | 6 | 13.0% (3.3-22.8) |
| Other | All Samples | 12 | 135 | 3.9% (2.4-5.4) |
|  | Senior National Women | 1 | 22 | 5.4% (3.2–7.6) |
|  | Senior National Men | 4 | 108 | 4.2% (0.7-7.8) |
|  | Senior Regional Men | 1 | 0 | 0.0% (-) |
|  | Senior Local Men | 1 | 0 | 0.0% (-) |
|  | Youth Regional Overall | 1 | 0 | 0.0% (-) |
|  | Concussion – Senior National Men | 1 | 1 | 2.9% (0-8.6) |
|  | Concussion – Youth Regional and Local Women | 1 | 1 | 4.8% (0-13.9) |
|  | Concussion – Youth Regional and Local Men | 1 | 2 | 4.3% (0-10.2) |
| Lineout | All Samples | 12 | 32 | 0.9% ((-)5.4-7.2) |
|  | Senior National Women | 1 | 3 | 0.7% (0–1.6) |
|  | Senior National Men | 4 | 19 | 0.7% (0.1-1.4) |
|  | Senior Regional Men | 1 | 9 | 33.3% (14.3–55.0) |
|  | Senior Local Men | 1 | 0 | 0.0% (-) |
|  | Youth Regional Overall | 1 | 1 | 1.4% (0.2-10) |
|  | Concussion – Senior National Men | 1 | 0 | 0.0% (-) |
|  | Concussion – Youth Regional and Local Women | 1 | 0 | 0.0% (-) |
|  | Concussion – Youth Regional and Local Men | 1 | 0 | 0.0% (-) |
| Maul | All Samples | 12 | 22 | 0.6% ((-)0.3-1.5) |
|  | Senior National Women | 1 | 0 | 0.0% (-) |
|  | Senior National Men | 4 | 9 | 0.4% (0.0-0.8) |
|  | Senior Regional Men | 1 | 0 | 0.0% (-) |
|  | Senior Local Men | 1 | 1 | 2.1% (3.0-11.0) |
|  | Youth Regional Overall | 1 | 0 | 0.0% (-) |
|  | Concussion – Senior National Men | 1 | 0 | 0.0% (-) |
|  | Concussion – Youth Regional and Local Women | 1 | 0 | 0.0% (-) |
|  | Concussion – Youth Regional and Local Men | 1 | 0 | 0.0% (-) |

Table 6: *cont.*

| **Match event** | **Sample classification** | **Number of samples** | **Specific injury count** | **Meta-analysed proportion (95% CI)** |
| --- | --- | --- | --- | --- |
| Scrum | All Samples | 12 | 22 | 0.6% (0.1-1.1) |
|  | Senior National Women | 1 | 1 | 0.2% (0–0.7) |
|  | Senior National Men | 4 | 14 | 0.5% ((-)0.1-1.2) |
|  | Senior Regional Men | 1 | 0 | 0.0% (-) |
|  | Senior Local Men | 1 | 1 | 2.1% (3.0-11.0) |
|  | Youth Regional Overall | 1 | 2 | 1.4% (0.2-10) |
|  | Concussion – Senior National Men | 1 | 0 | 0.0% (-) |
|  | Concussion – Youth Regional and Local Women | 1 | 0 | 0.0% (-) |
|  | Concussion – Youth Regional and Local Men | 1 | 0 | 0.0% (-) |
| Kicking | All Samples | 12 | 18 | 0.5% ((-)0.3-1.3) |
|  | Senior National Women | 1 | 4 | 1.0% (0.0–1.9) |
|  | Senior National Men | 4 | 12 | 0.5% ((-)0.2-1.1) |
|  | Senior Regional Men | 1 | 0 | 0.0% (-) |
|  | Senior Local Men | 1 | 2 | 4.3% (1.0-14.0) |
|  | Youth Regional Overall | 1 | 0 | 0.0% (-) |
|  | Concussion – Senior National Men | 1 | 0 | 0.0% (-) |
|  | Concussion – Youth Regional and Local Women | 1 | 0 | 0.0% (-) |
|  | Concussion – Youth Regional and Local Men | 1 | 0 | 0.0% (-) |

*Note: N/A = not applicable.*

**SUPPLEMENTARY MATERIAL 11**

Table 7: Injury event – Match injuries as a function of type of contact during injury. The meta-analysis combined all proportions from the respective studies.

| **Contact type** | **Sample classification** | **Number of samples** | **Specific injury count** | **Meta-analysed proportion (95% CI)** |
| --- | --- | --- | --- | --- |
| Contact | All Samples | 21 | 3242 | 78.4% (74.1-82.6) |
|  | Senior National Women | 4 | 479 | 89.1% (80.0-98.1) |
|  | Senior National Men | 7 | 2236 | 77.4% (73.8-81.1) |
|  | Senior Regional Men | 1 | 22 | 81.5% (65.5-93.7) |
|  | Senior Local Men | 1 | 34 | 72.9% (59.0-83.0) |
|  | Youth National Overall | 2 | 54 | 73.4% (58.2-88.7) |
|  | Youth Regional Overall | 1 | 56 | 81.2% (69.9-88.9) |
|  | Concussion – Senior National Men | 1 | 34 | 100% (-) |
|  | Concussion – Youth Regional and Local Women | 1 | 21 | 100% (-) |
|  | Concussion – Youth Regional and Local Men | 1 | 46 | 100% (-) |
| Non-contact | All Samples | 21 | 775 | 18.7% (14.9-22.5) |
|  | Senior National Women | 4 | 59 | 10.9% (1.9-20.0) |
|  | Senior National Men | 7 | 650 | 22.5% (18.9-26.1) |
|  | Senior Regional Men | 1 | 5 | 18.5 (N/R) |
|  | Senior Local Men | 2 | 15 | 24.1% (7.4-40.7) |
|  | Youth National Overall | 2 | 8 | 10.5% ((-)18.0-39.1) |
|  | Youth Regional Overall | 1 | 4 | 5.8% (2.1-4.8) |
|  | Concussion – Senior National Men | 1 | 0 | 0.0% (-) |
|  | Concussion – Youth Regional and Local Women | 1 | 0 | 0.0% (-) |
|  | Concussion – Youth Regional and Local Men | 1 | 0 | 0.0% (-) |

*Note: N/R = not reported within the study.*

**SUPPLEMENTARY MATERIAL 12**

Table 8: Injury event – Match injuries as a function of the players’ positions. The meta-analysis combined all proportions from the respective studies.

| **Injury Type** | **Sample Classification** | **Number of samples** | **Specific injury count** | **Meta-analysed proportion (95% CI)** |
| --- | --- | --- | --- | --- |
| Forwards | All Samples | 13 | 1483 | 42.2% (33.6-50.7) |
|  | Senior National Women | 1 | 150 | 36.1% (N/R) |
|  | Senior National Men | 4 | 1164 | 45.0% (25.2-64.9) |
|  | Senior Regional Men | 2 | 18 | 40.0% (28.5-51.4) |
|  | Senior Local Men | 1 | 14 | 29.2% (N/R) |
|  | Youth Regional Overall | 1 | 16 | 23.2% (N/R) |
|  | Concussion – Senior National Men | 1 | 13 | 38.2% (N/R) |
|  | Concussion – Youth Regional and Local Women | 1 | 7 | 33.3% (N/R) |
|  | Concussion – Youth Regional and Local Men | 1 | 10 | 21.1% (N/R) |
| Backs | All Samples | 13 | 2009 | 57.1% (45.6-68.7) |
|  | Senior National Women | 1 | 266 | 63.9% (N/R) |
|  | Senior National Men | 4 | 1417 | 54.8% (35.7-73.9) |
|  | Senior Regional Men | 2 | 27 | 60.0% (57.5-62.5) |
|  | Senior Local Men | 1 | 31 | 66.7% (N/R) |
|  | Youth Regional Overall | 1 | 45 | 65.2% (N/R) |
|  | Concussion – Senior National Men | 1 | 21 | 61.8% (N/R) |
|  | Concussion – Youth Regional and Local Women | 1 | 14 | 66.3% (N/R) |
|  | Concussion – Youth Regional and Local Men | 1 | 36 | 78.3% (N/R) |

*Note: N/R = not reported within the study.*

**SUPPLEMENTARY MATERIAL 13**

Table 9: Injury event – Match injuries as a function of the period of the match the injury occurred. The meta-analysis combined all proportions from the respective studies.

| **Injury Type** | **Sample Classification** | **Number of samples** | **Specific injury count** | **Meta-analysed proportion (95% CI)** |
| --- | --- | --- | --- | --- |
| First half | All Samples | 11 | 1303 | 39.7% (34.6-44.9) |
|  | Senior National Women | 1 | 175 | 42.1% (37.3-46.9) |
|  | Senior National Men | 4 | 924 | 37.9% (32.2-43.7) |
|  | Senior Regional Men | 1 | 16 | 59.3% (41.4-76.0) |
|  | Senior Local Men | 1 | 22 | 47.6% (N/R) |
|  | Concussion – Senior National Men | 1 | 18 | 52.9% (36.2-69.7) |
|  | Concussion – Regional and Local Women | 1 | 10 | 46.0% (N/R) |
|  | Concussion – Regional and Local Men | 1 | 21 | 46.0% (N/R) |
| Second half | All Samples | 11 | 1976 | 60.3% (55.0-65.5) |
|  | Senior National Women | 1 | 241 | 57.9% (53.1-62.7) |
|  | Senior National Men | 4 | 1511 | 62.1% (56.7-67.4) |
|  | Senior Regional Men | 1 | 11 | 40.7% (N/R) |
|  | Senior Local Men | 1 | 25 | 52.4% (N/R) |
|  | Concussion – Senior National Men | 1 | 16 | 47.1% (30.3-63.8) |
|  | Concussion – Youth Regional and Local Women | 1 | 11 | 54.0% (N/R) |
|  | Concussion – Youth Regional and Local Men | 1 | 25 | 54.0% (N/R) |

*Note: N/R = not reported within the study.*

**SUPPLEMENTARY MATERIAL 14**

Table 10: Description of studies that reported risk factors associated with injury.

| **Study** | **Risk Factors Reported** | **Dependent Variable/Outcome Measure** | **Quality of Study** | **Type of Statistical Analysis** |
| --- | --- | --- | --- | --- |
| Fuller & Taylor (38) | Position, age, height, weight | Number of injuries | 9 | Unpaired T-tests |
| Lopez et al. (24) | Position, age, height, weight | Number of injures | 8 | T-test & x^2^ tests |
|  | Playing surfaces | Injury incidence rate |  | Rate ratios |
|  | Protective equipment use | Number of injuries |  | X^2^ tests |
| Fuller, Taylor & Raftery (33) | Position, age, height, weight | Number of concussions | 9 | T-tests |
|  | Order of tournament in the series | Concussion incidence rate |  | Z-tests |
|  | Order of tournament in the series | Concussion severity |  | Z-tests |
|  | Player in starting line-up | Number of concussions |  | X^2^ test |
|  | Order of tournament in the series | Number of players returned to play <7 days |  | X^2^ tests |
| Cruz-Ferreira et al. (28) | Position, age, height, weight, playing experience | Number of injuries & Injury incidence rates | 7 | T-tests |
| Fuller, Taylor & Raftery (31) | Day of the tournament | Number of injuries | 9 | X^2^ tests |
|  | Match half by day of tournament | Proportion of injuries |  | Z-tests |
| Xu et al. (32) | Matches per tournament | Injury incidence rate | 5 | N/A |
|  | Day of the tournament | Injury incidence rate |  | N/A |
|  | Weather per tournament | Injury incidence |  | N/R |
| Lopez et al. (23) | Match number of the tournament | Concussion proportion | 9 | X^2^ tests |
|  | Match number of the tournament | Concussion severity |  | X^2^ tests |
|  | Number of re-injuries | Proportion of injuries |  | N/A |
|  | Number of subsequent concussions | Injury severity |  | X^2^ tests |
|  | Protective equipment use | Number of injuries |  | X^2^ tests |
| Fuller, Taylor & Raftery (35) | Order of tournament in the series | Injury incidence rate | 9 | Z-tests |
|  | Distance travelled pre-tournament | Number of injuries |  | X^2^ tests |
|  | Distance travelled pre-tournament | Number of injuries |  | X^2^ tests |
|  | Distance travelled pre-tournament | Injury proportion by location, type, nature of event, and mechanism of injury |  | Z-tests |
|  | Distance travelled pre-tournament | Injury incidence rate |  | Z-tests |
|  | Distance travelled pre-tournament | Injury severity (mean) |  | Z-tests |
|  | Distance travelled pre-tournament | Injury severity (median) |  | Mann-Whitney U tests |
| Rizi et al. (29) | Time to onset of injury | Categorical injury severity | 5 | X^2^ tests |
|  | Previous injury, gender, age, playing experience, weight, height, and physical test data | Categorical injury severity |  | Cox proportional hazards regression model |
| Ma et al. (25) | Match number of the tournament | Proportion of injuries | 9 | N/A |
|  | Playing surfaces | Injury incidence rate |  | Rate ratios |
|  | Protective equipment use | Number of injuries |  | X^2^ tests |
| Toohey et al. (26) | Number of subsequent injuries | Proportion of players | 8 | N/A |
|  | Number of injury free days | Number of subsequent injuries |  | N/A |

*Note: N/A = not applicable to the study, N/R = not reported.*

**SUPPLEMENTARY MATERIAL 15**

Articles excluded at full-text review:

| **No.** | **Article Name** | **Authors** | **Year** | **Reason for exclusion** |
| --- | --- | --- | --- | --- |
| 1. | An American experience with a new Olympic collision sport: Rugby sevens | Lopez, et al. | 2014 | Conference abstract with no link to full-text paper |
| 2. | The Epidemiology of Concussive Injuries in Rugby-7s: An American Experience | Lopez, et al. | 2015 | Conference abstract with no link to full-text paper |
| 3. | Injuries in Elite U.S. Rugby 7s Tournament Players over 4 years: Time Loss and Medical Attention | Lopez, et al. | 2016 | Conference abstract with no link to full-text paper |
| 4. | Injury Profile Of Time-loss Injuries In Non-elite/community U.S. Rugby-7s Tournament Players | Ma et al. | 2016 | Conference abstract with no link to full-text paper |
| 5. | Same, same but different workload risk profiles for men and women in rugby sevens | Drew et al. | 2017 | Conference abstract with no link to full-text paper |
| 6. | Subsequent injury in rugby sevens – More than just recurrence! | Toohey et al. | 2017 | Conference abstract with no link to full-text paper |
| 7. | An Epidemiological Injury Profile Of Sub-elite U.S. Rugby-7s (USA Rugby National Club Championships) | Lopez, et al. | 2017 | Conference abstract with no link to full-text paper |
| 8. | Injury rates of U.S. rugby-7s an Olympic collision sport: Using a novel injury surveillance tool the RISE report methodology | Lopez et al. | 2017 | Conference abstract with no link to full-text paper |
| 9. | US Rugby-7s Injuries in Levels Of Play: A 5-year Epidemiological Prospective Study Of An Emerging Olympic Collision Sport In A Developing Market | Victoria et al. | 2017 | Conference abstract with no link to full-text paper |
| 10. | Profile Of Non-time-loss Conditions/Injuries in U.S. Men’s Rugby-7s Players | Ma et al. | 2018 | Conference abstract with no link to full-text paper |
| 11. | A Seven-year Epidemiological Analysis Of Ankle Injuries In U.S. Rugby-7s | Lopez et al. | 2019 | Conference abstract with no link to full-text paper |
| 12. | Gender Differences In The Risk Of Head, Neck & Face Injuries In US Rugby-7s | Victoria et al. | 2019 | Conference abstract with no link to full-text paper |
| 13. | Gender Differences In Match Contact Injuries In U.S. Rugby-7 | Ma et al. | 2019 | Conference abstract with no link to full-text paper |
| 14. | "Recognise and Remove": A universal principle for the management of sports injuries | Fuller | 2018 | Data reported from previous published data |
| 15. | Psychological demands of international rugby sevens and well-being needs of elite South African players | Kruyt & Grobbelaar | 2019 | No injury or illness epidemiological data |
| 16. | The Frequency and Severity of Gastrointestinal Symptoms in Rugby Players | Chantler et al. | 2024 | No injury or illness incidence rates reported |
| 17. | Breast injuries reported by female contact football players based on football code, player position and competition level | Brisbine et al. | 2020 | No sevens cohort exclusively reported |
| 18. | The burden of match injuries in Portuguese senior male club rugby union: a cohort prospective study | Miguel et al. | 2023 | No sevens cohort exclusively reported |
| 19. | Collapsed scrums and collision tackles: What is the injury risk? | Roberts et al. | 2015 | No sevens cohort exclusively reported |
| 20. | Preseason Functional Movement Screen Component Tests Predict Severe Contact Injuries in Professional Rugby Union Players | Tee et al. | 2016 | No sevens cohort exclusively reported |
| 21. | Injury Patterns, Physiological Profile, and Performance in University Rugby Union | Ball et al. | 2018 | No sevens cohort exclusively reported |
| 22. | Collision and Contact Sport Participation and Quality of Life Among Adolescent Athletes | Howell et al. | 2020 | No sevens cohort exclusively reported |
| 23. | Movement Demands and Injury Characteristics in Under-20-Years University Rugby Union Players | Ball, Halaki & Orr | 2020 | No sevens cohort exclusively reported |
| 24. | The prevalence of stress urinary incontinence among elite female rugby union players in Australia | Faulks & Catto | 2021 | No sevens cohort exclusively reported |
| 25. | Single season re-injury risk after concussion and lower extremity injury among male, collision sport, high school athletes | Wilson et al. | 2021 | No sevens cohort exclusively reported |
| 26. | Epidemiology of rugby-related fractures in high school- and college-aged players in the United States: an analysis of the 1999–2018 NEISS database | Etzel, et al. | 2022 | No sevens cohort exclusively reported |
| 27. | Injuries in Canadian high school boys’ collision sports: insights across football, ice hockey, lacrosse, and rugby | West et al. | 2023 | No sevens cohort exclusively reported |
| 28. | Prevalence of Staphylococcus aureus carriage and pattern of antibiotic resistance, including methicillin resistance, among contact sport athletes in Italy | Mascaro et al. | 2019 | No sevens cohort exclusively reported, and no injury or illness incidence rates reported |
| 29. | Incidence of injury and its prevention in the elite series of women in rugby sevens in Czech Republic | Pravečková et al. | 2019 | Non-english paper |
| 30. | Traumatology in rugby sevens | Kaux et al. | 2014 | Non-english paper |
| 31. | Epidemiological study of injuries in international Rugby Sevens | Fuller, Taylor & Molloy | 2010 | Included in previous systematic review |
| 32. | Profile of an American amateur rugby union sevens series | Lopez et al | 2012 | Included in previous systematic review |
| 33. | Epidemiology of concussion in men's elite Rugby-7s (Sevens World Series) and Rugby-15s (Rugby World Cup, Junior World Championship and Rugby Trophy, Pacific Nations Cup and English Premiership) | Fuller, Taylor & Raftery | 2015 | Included in previous systematic review |
